# Navigating the risk of living donor liver transplantation from older donors: analysis of 4035 cases from a multicenter cohort

**DOI:** 10.1097/JS9.0000000000003522

**Published:** 2025-09-22

**Authors:** Eun-Ki Min, Jae Geun Lee, YoungRok Choi, Jong Man Kim, Young Kyoung You, Donglak Choi, Je Ho Ryu, Bong-Wan Kim, Dong-Sik Kim, Jai Young Cho, Yang Won Nah, Man Ki Ju, Koo Jeong Kang, Soo Jin Na Choi, Shin Hwang, Deok-Gie Kim

**Affiliations:** a Department of Surgery, The Research Institute for Transplantation, Yonsei University College of Medicine, Seoul, South Korea; b Department of Surgery, Seoul National University College of Medicine, Seoul, South Korea; c Department of Surgery, Samsung Medical Center, Sungkyunkwan University School of Medicine, Seoul, South Korea; d Department of Surgery, College of Medicine, The Catholic University of Korea, Seoul, South Korea; e Department of Surgery, Catholic University of Daegu, Daegu, South Korea; f Department of Surgery, Pusan National University Yangsan Hospital, Pusan National University School of Medicine, Pusan, South Korea; g Department of Liver Transplantation and Hepatobiliary Surgery, Ajou University School of Medicine, Suwon, South Korea; h Department of Surgery, Korea University College of Medicine, Seoul, South Korea; i Department of Surgery, Seoul National University Bundang Hospital, Seongnam, South Korea; j Department of Surgery, Ulsan University Hospital, University of Ulsan College of Medicine, Ulsan, South Korea; k Department of Surgery, Gangnam Severance Hospital, Yonsei University College of Medicine, Seoul, South Korea; l Department of Surgery, Dongsan Medical Center, Keimyung University School of Medicine, Daegu, South Korea; m Division of Transplant & Vascular Surgery, Department of Surgery, Chonnam National University Hospital, Gwangju, South Korea; n Department of Surgery, College of Medicine University of Ulsan, Asan Medical Center, Seoul, South Korea

**Keywords:** elderly donor, graft survival, KOTRY, living donor liver transplantation, old donor

## Abstract

**Introduction:**

Ensuring both graft survival and donor safety with old liver donors remains an important issue in living donor liver transplantation (LDLT). This study aimed to evaluate recipient and donor outcomes based on donor age and to identify risk factors associated with graft loss in LDLT involving old donors.

**Materials and methods:**

We included 4035 LDLT cases from a multicenter cohort, categorizing donors as “old” or “young” based on an age cutoff of 50 years determined by a restricted cubic spline. After 1:3 propensity score matching, graft survival was compared between the groups. Risk factors for graft loss in old versus young donor LDLT were investigated through interaction analysis, with outcomes stratified by the number of risk factors.

**Results:**

The old-donor group (*n* = 374; 9.3%) showed significantly lower 5-year graft survival than the young-donor group (*n* = 3661; 90.7%) in the matched cohort (79.6% vs. 87.7%, *P* = 0.004). Old-donor was an independent risk factor for graft loss [adjusted hazard ratio (HR): 1.56, 95% CI: 1.17–2.07, *P* = 0.002]. Significant interactions affecting graft survival in old-donor LDLT compared to young-donor LDLT included cold ischemic time (CIT) ≥ 150 min, Model for End-stage Liver Disease (MELD) score ≥ 20, and recipient BMI ≥ 25 kg/m^2^. Old-donor LDLT with two or more of these risk factors increased the risk of graft loss (HR 3.78, 95% CI: 1.97–7.26, *P* < 0.001). Six-month donor complication rates did not differ by age (*P* = 0.672).

**Conclusions:**

LDLT using grafts from old donors (≥50 years) showed poorer graft survival, especially when two or more of the following were present: CIT ≥ 150 min, MELD ≥ 20, or recipient BMI ≥ 25 kg/m^2^. These risk factors should be carefully considered when selecting older living donors.

## Graphical abstract

**Figure:**
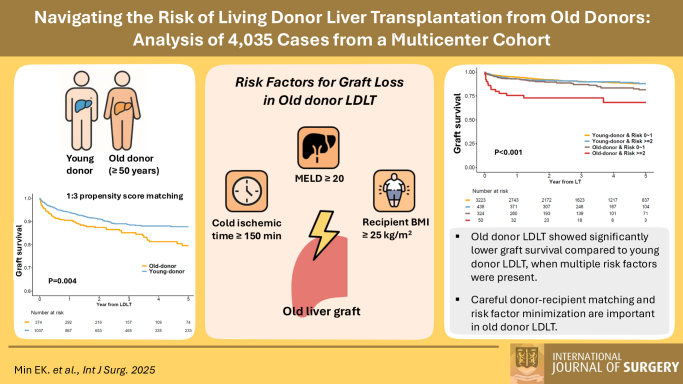

**HIGHLIGHTS**

- Although the feasibility of living donor liver transplantation (LDLT) using older living donors has been reported, data from large-volume cohorts remain limited, and comparative analyses of relative risk factors versus younger donors are lacking.
- In this multicenter study containing 4035 adult LDLT, older donor (≥50 years) grafts were associated with poorer 5-year graft survival compared to younger donor (<50 years), especially when ≥2 risk factors – cold ischemic time ≥ 150 min, MELD ≥ 20, or BMI ≥ 25 kg/m^2^ – were present.
- These risk factors should be carefully evaluated to ensure the safe selection of older living donors in LDLT.

## Introduction

Living donor liver transplantation (LDLT) has been adopted worldwide as a feasible option to mitigate the waitlist mortality resulting from the shortage of deceased donor organs^[1]^. However, given the limited availability of living donors, expanding the donor pool while ensuring favorable outcomes for both donors and recipients continues to be a significant focus in LDLT^[2]^.

The regenerative capacity of partial liver grafts is crucial for restoring function and ensuring graft survival after LDLT^[3]^. This raises concerns regarding old donors, as aged liver show a decline in regenerative potential^[4,5]^. A recent analysis of the United Network for Organ Sharing database identified advanced living donor age as a significant risk factor for reduced recipient and graft survival^[6]^. Similarly, studies from Japan have reported a significant association between increasing donor age and poorer recipient outcomes in LDLT^[7–10]^. However, some institutions have reported acceptable outcomes in LDLT using old living donors through careful donor-recipient selection, with varying age cutoffs ranging from 50 to 60 years^[11–14]^. Furthermore, a recent meta-analysis reported that LDLT using donors aged over 50 showed comparable survival outcomes to those using younger donors^[15]^.

Nevertheless, previous studies often failed to adequately control differences in characteristics between age groups^[13,14]^, leading to insufficient evidence on the safety of LDLT with old donors. Additionally, the reported acceptable outcomes may largely reflect the effects of meticulous donor-recipient selection according to each center’s policy [e.g., including only grafts with a graft-to-recipient weight ratio (GRWR) ≥0.8 or recipients with low Model for End-Stage Liver Disease (MELD) scores]^[11,12]^. Moreover, single-center studies with small sample sizes limit many analysis^[7,9–13]^, and a definitive age cutoff for old donors is lacking. Importantly, there is limited research identifying specific factors that increase the risk of graft loss in old donor LDLT compared to younger donor LDLT.

Therefore, using data from a Korean multicenter registry, we aimed to evaluate recipient and donor outcomes based on donor age and identify key risk factors associated with LDLT from old donors through interaction analysis.

## Materials and methods

This retrospective study adhered to the strengthening the reporting of cohort, cross-sectional, and case–control studies in surgery (STROCSS) criteria^[16]^ (Supplemental Digital Content 1, available at: http://links.lww.com/JS9/F186), and the principles outlined in the Declarations of Helsinki and Istanbul. Approval for the overall multicenter registry has been detailed in a previous publication^[17]^.

## Study cohort description

This research used a multicenter retrospective cohort design encompassing a sample of 4656 individuals who underwent LDLT, as cataloged in the Korean Organ Transplantation Registry (KOTRY) from May 2014 through December 2021. The KOTRY database is continuously managed, and its data collection methodology has been documented elsewhere^[17]^. Exclusions included individuals aged 18 years or younger (*n* = 95), patients with non-HCC liver malignancies (*n* = 73), recipients of grafts from multiple donors (*n* = 48), retransplantation cases (*n* = 24), and incomplete records (*n* = 51). Finally, 4035 LDLT recipients were analyzed (Supplemental Digital Content Figure S1, available at: http://links.lww.com/JS9/F186).

## Data acquisition and definitions

Comprehensive data on recipients, donors, and surgical procedures were obtained from the KOTRY database. The etiology of liver disease in recipients was categorized as viral, alcoholic, or other causes. Grafts were primarily classified based on the lobe used, predominantly the right lobe, versus others, mainly the left lobe. Graft steatosis was grouped based on the fat content exceeding or not exceeding 10%, confirmed via pathology at donor surgery. GRWR was quantified by the formula: GRWR = [graft weight (g)/recipient weight (g)] × 100. Cold ischemic time (CIT) was defined as the duration from graft inflow clamping in the donor to the time of reperfusion. The study’s primary endpoint was graft loss, defined as the need for re-transplantation or patient death. The follow-up period extended up to 5 years post-transplant or until December 2022, whichever occurred first.

## Propensity score matching

Individuals from the old-donor and young-donor groups were matched in a 1:3 ratio using propensity scores derived from all baseline variables. Matching utilized the nearest neighbor method with a caliper width set at 0.1^[18]^, ensuring standardized mean differences <0.1 between the two groups. Matches not meeting this criterion were excluded.

## Relative risk factors for graft loss using old-donor versus young-donor

In the unmatched population, risk factors for graft loss with old-donor versus young-donor were explored through subgroup analysis using multivariable Cox regression. Continuous variables were categorized by the cutoff where the unadjusted hazard of old donor versus young donor was significant, if applicable. Otherwise, a cutoff was applied where the hazard for graft loss starts to increase, or a traditional cutoff was used for subgroup analysis. The principles for categorizing each variable are provided in Supplemental Digital Content Table S2, available at: http://links.lww.com/JS9/F186, Supplemental Digital Content 1, available at: http://links.lww.com/JS9/F185.

The conditions for selecting subgroups as specific risk factors for old-donor were similar to our previous study^[19]^: (1) the hazard ratio (HR) for graft survival indicated a significant difference in one subgroup with old-donor grafts compared to its counterpart, where no significant difference was observed, and (2) the interaction *P*-value for the correlation between the donor age group and the subgroup factor was <0.1. Additionally, we analyzed how the accumulation of these risk factors affects the hazard relating to graft loss in the comparison of old-donor and young-donor groups.

## Statistical analysis

Data were reported as median [interquartile range (IQR)] or as counts (percentage), depending on the variable type. Comparisons between continuous and categorical variables were made using the Student’s t-test or chi-square test as required. Graft survival among propensity score-matched cohorts was evaluated using Kaplan–Meier survival plots and the log-rank test. Variables showing *P*-values below 0.1 in univariable Cox regression were included in multivariable Cox regression models to determine independent predictors of graft loss in old-donor versus young-donor groups, calculating HRs for graft loss. All subgroup and comprehensive analysis were adjusted for the same covariates as in the fully eligible cohort. These statistical analyses were executed using R software, version 4.4.0 on macOS (http://cran.r-project.org/), with significance set at *P* < 0.05.

## Results

### Age distribution and categorization of living donors

The age distribution of living donors is depicted in Supplemental Digital Content Figure S2A, available at: http://links.lww.com/JS9/F186, Supplemental Digital Content 1, available at: http://links.lww.com/JS9/F185. The median donor age was 30 years (IQR: 24–39; minimum, 16; maximum, 71). The donor age cutoff was determined using a smoothing spline curve, adjusted for all independent risk factors associated with graft loss (Supplemental Digital Content Figure S2B, available at: http://links.lww.com/JS9/F186; Supplemental Digital Content 1, available at: http://links.lww.com/JS9/F185). The population was divided into old-donor (*n* = 374) and young-donor (*n* = 3661) groups using a donor cutoff age of 50 years. In the entire population, graft survival stratified by 10-year donor age intervals revealed superior graft survival for donors aged <20, 20–29, and 30–39 years, while the poorest outcomes were observed for donors aged ≥60 years (Supplemental Digital Content Figure S3, available at: http://links.lww.com/JS9/F186; Supplemental Digital Content 1, available at: http://links.lww.com/JS9/F185).

### Baseline characteristics

In the entire cohort, the recipient age was slightly younger in the old-donor group than the young-donor group [56 (51–60) vs. 56 (51–61) years, *P* = 0.049]. The proportion of males (70.1% vs. 71.7%, *P* = 0.540) and body mass index [BMI; 23.5 (21.5–25.9) vs. 23.9 (21.7–26.2) kg/m^2^, *P* = 0.143] were similar (Table 1). A higher proportion of LTs was found in the recent era (2018–2020) in the old-donor group (65.5% vs. 59.4%, *P* = 0.025). HCC prevalence (56.4% vs. 54.8%, *P* = 0.585) and MELD score [12 (8–18) vs. 12 (9–18), *P* = 0.347] were comparable. ABO-incompatible LTs (30.2% vs. 22.6%, *P* = 0.001) were more frequent in the old-donor group, while male donors were less frequent (34.8% vs. 66.1%, *P* < 0.001). Donor BMI was higher in the old-donor group [23.8 (22.0–25.5) vs. 23.4 (21.4–25.4) kg/m^2^, *P* = 0.039]. GRWR < 0.8 was more frequent in the old-donor group (15.0% vs. 9.6%, *P* = 0.001), while use of non-right lobe grafts (5.1% vs. 4.9%, *P* = 0.952) and graft steatosis >10% (14.7% vs. 11.2%, *P* = 0.053) showed no between-group differences. CIT was longer in the old-donor group [90.0 (72.0–126.0) vs. 90.0 (66.0–114.0) min, *P* = 0.012].

**Table 1:** Baseline characteristics of the study participants

Variables	Old-donor (*n* = 374)	Young-donor (*n* = 3661)	*P*	Young-donor, matched (*n* = 1037)	SMD
Age, years	56 (51–60)	56 (51–61)	0.049	56 (49–61)	−0.027
Sex, male	262 (70.1)	2625 (71.7)	0.540	712 (68.7)	0.033
BMI, kg/m^2^	23.5 (21.5–25.9)	23.9 (21.7–26.2)	0.143	23.6 (21.6–25.9)	0.006
Year of LT			0.025		−0.020
2014–2017	129 (34.5)	1487 (40.6)		353 (34.0)	
2018–2020	245 (65.5)	2174 (59.4)		684 (66.0)	
Underlying liver disease			0.587		−0.022
Viral	235 (62.8)	2220 (60.6)		658 (63.5)	
Alcohol	85 (22.7)	920 (25.1)		235 (22.7)	
Others	54 (14.4)	521 (14.2)		144 (13.9)	
Diabetes mellitus	100 (26.7)	1040 (28.4)	0.533	290 (28.0)	−0.013
HCC	211 (56.4)	2006 (54.8)	0.585	596 (57.5)	−0.026
Refractory ascites	83 (22.2)	753 (20.6)	0.502	230 (22.2)	0.006
Encephalopathy	47 (12.6)	348 (9.5)	0.071	132 (12.7)	−0.020
Hospitalization			<0.001		0.010
OPD	244 (65.2)	1649 (45.0)		646 (62.3)	
Ward	115 (30.7)	1897 (51.8)		347 (33.5)	
ICU	15 (4.0)	115 (3.1)		44 (4.2)	
Pre-transplant MELD	12 (8–18)	12 (9–18)	0.347	12 (9–18)	0.011
ABO incompatibility	113 (30.2)	827 (22.6)	0.001	291 (28.1)	0.010
Donor sex, male	130 (34.8)	2420 (66.1)	<0.001	389 (37.5)	0.024
Donor BMI, kg/m^2^	23.8 (22.0–25.5)	23.4 (21.4–25.4)	0.039	23.4 (21.2–25.7)	0.011
GRWR < 0.8	56 (15.0)	352 (9.6)	0.001	151 (14.6)	−0.010
Graft type			0.952		−0.030
Right	319 (85.3)	3251 (88.8)		975 (94.0)	
Other than right	19 (5.1)	178 (4.9)		62 (6.0)	
Graft steatosis >10%	55 (14.7)	410 (11.2)	0.053	143 (13.8)	0.009
Cold ischemic time, min	90.0 (72.0–126.0)	90.0 (66.0–114.0)	0.012	90.0 (66.0–120.0)	−0.061

SMD, standardized mean difference; BMI, body mass index; LT, liver transplantation; HCC, hepatocellular carcinoma; OPD, outpatient department; ICU, intensive care unit; MELD, model for end stage liver disease; GRWR, graft-to-recipient weight ratio.

After 1:3 propensity matching, 374 old-donor LDLT cases were matched to 1037 young-donor LDLT cases, with all characteristics well-balanced between the two groups (standardized mean difference <0.1). The entire study population is summarized in Supplemental Digital Content Figure S1, available at: http://links.lww.com/JS9/F186, Supplemental Digital Content 1, available at: http://links.lww.com/JS9/F185.

### Graft survival between groups and multivariable cox analysis

**Figure 1.**
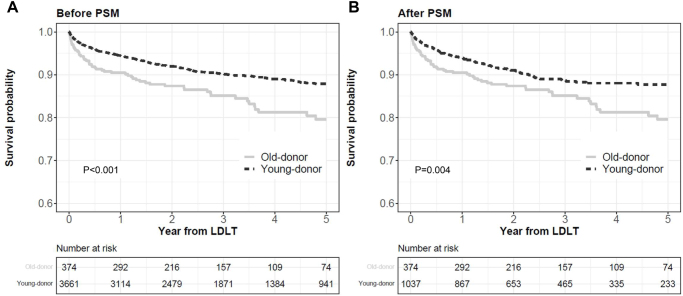
Comparison of graft survival before and after propensity score matching. Graft survival in the old-donor group is significantly lower than that in the young-donor group in both the entire cohort (A) and the matched cohort (B). PSM, propensity score matching; LDLT, living donor liver transplantation.

In both the entire and matched cohorts, graft survival in the old-donor group was significantly lower than in the young-donor group (*P* < 0.001 and *P* = 0.004, Fig. 1A,B). In the matched cohort, the respective graft survival rates at 1, 3, and 5 years were 90.5%, 85.1%, and 79.6% in the old-donor group, and 94.0%, 88.7%, and 87.7% in the young-donor group. Multivariable Cox regression analysis found that the old-donor group was significantly associated with a higher rate of graft loss (HR: 1.56, *P* = 0.002) (Supplemental Digital Content Table S1, available at: http://links.lww.com/JS9/F186; Supplemental Digital Content 1, available at: http://links.lww.com/JS9/F185). The most common cause of death was graft loss in the old-donor group (31.1%), whereas infection in the young-donor group (31.7%). Among graft loss, the proportion of primary non-function was 14% in the old-donor group and 5.6% in the young-donor group (Supplemental Digital Content Table S2, available at: http://links.lww.com/JS9/F186)

### Risk factors for graft loss in old-donor LDLT

**Figure 2.**
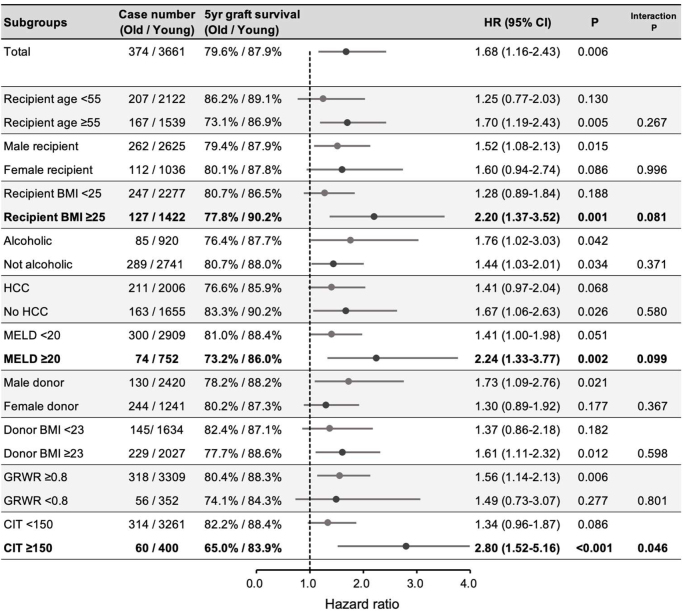
Subgroup analysis to determine the relative risk factors for old-donor LDLT. Variables highlighted in bold indicate the risk factors for old-donor LDLT. These risk factors were variables for which the interaction *P*-value between the variable subgroup and the old-donor group was <0.1. LDLT, living donor liver transplantation; BMI, body mass index; MELD, model for end-stage liver disease; GRWR, graft-to-recipient weight ratio; CIT, cold ischemic time (in min).

As shown in Figure 2, subgroup analysis according to variables with each cutoff (Supplemental Digital Content Table S3, available at: http://links.lww.com/JS9/F186; Supplemental Digital Content 1, available at: http://links.lww.com/JS9/F185) identified three risk factors for graft loss in old-donor LDLT: CIT ≥ 150 min (HR: 2.80, *P* < 0.001), MELD score ≥ 20 (HR: 2.24, *P* < 0.002), and recipient BMI ≥ 25 kg/m^2^ (HR: 2.20, *P* < 0.001) (Supplemental Digital Content Figures S5–S7, available at: http://links.lww.com/JS9/F186; Supplemental Digital Content 2, available at: http://links.lww.com/JS9/F186).

The relative risk for graft loss in old-donor LDLT against young-donor LDLT was significant for donor BMI ≥ 23 kg/m^2^ (Supplemental Digital Content Figure S8, available at: http://links.lww.com/JS9/F186; Supplemental Digital Content 2, available at: http://links.lww.com/JS9/F186). However, this factor was not included as the final risk factor after interaction analysis (interaction *P* = 0.598). The HR for graft loss in old-donor LDLT did not differ significantly for GRWR < 0.8 (Supplemental Digital Content Figures S9, S10, available at: http://links.lww.com/JS9/F186; Supplemental Digital Content 2, available at: http://links.lww.com/JS9/F186), nor graft steatosis >10% (Supplemental Digital Content Figure S11, available at: http://links.lww.com/JS9/F186; Supplemental Digital Content 2, available at: http://links.lww.com/JS9/F186).

### Risk of graft loss, relative to the number of risk factors

In the Kaplan–Meier survival analysis, old-donor LDLT procedures with two or more risk factors (CIT ≥ 150 min, MELD score ≥ 20, recipient BMI ≥ 25 kg/m^2^) had significantly lower 5-year graft survival rates compared to those with no or one risk factor. This pattern was not observed in the young-donor group (Supplemental Digital Content Figure S12, available at: http://links.lww.com/JS9/F186; Supplemental Digital Content 2, available at: http://links.lww.com/JS9/F186).

Adjusted HRs for graft loss showed no significant difference between old- and young-donor LDLT in recipients with no (HR: 1.24, *P* = 0.391) or one risk factor (HR: 1.46, *P* = 0.092) (Table 2). However, in cases with two or more risk factors, the risk of graft loss was significantly higher in old-donor LDLT (HR: 3.78, *P* < 0.001). Significantly poorer graft survival of old-donor LDLT accompanying two or more risk factors compared to those with no or only one risk factor or to young-donor LDLT was particularly pronounced within the first 6 months post-LT (Supplemental Digital Content Figure S13, available at: http://links.lww.com/JS9/F186; Supplemental Digital Content 2, available at: http://links.lww.com/JS9/F186).

**Table 2:** Hazard ratio for graft loss of the old-donor versus young-donor groups according to the number of risk factors

	Case number (old/young)	5-year survival (old/young)	Adjusted HR^a^	95% CI	*P*
No risk factor	168/1599	82.7%/87.8%	1.24	0.76–2.01	0.391
1 risk factor	156/1624	80.2%/87.9%	1.46	0.94–2.28	0.092
≥2 risk factors	50/438	68.1%/87.9%	3.78	1.97–7.26	<0.001

HR, hazard ratio; CI, confidence interval.

Risk factors included recipient BMI ≥ 25 kg/m^2^, MELD ≥ 20, and CIT ≥ 150 min, which were identified as significant risk factors using subgroup analysis.

^a^ HRs were adjusted using the same covariates as in the multivariable Cox models for the entire population.

### Recipient post-LT complications according to donor age group

As presented in Supplemental Digital Content Table S4, available at: http://links.lww.com/JS9/F186, recipients in the old-donor group experienced a higher rate of biliary complications compared to those in the young-donor group (43.9% vs. 27.8%, aHR 1.41, *P* < 0.001). In contrast, hepatic artery, portal vein, and hepatic vein complications were comparable between the two groups. The rate of biopsy-proven rejection was also significantly higher in the old-donor group (18.4% vs. 7.5%, aHR 1.63, *P* = 0.009). However, among recipients in the old-donor group, neither biliary complications nor rejection were associated with graft loss (Supplemental Digital Content Table S5, available at: http://links.lww.com/JS9/F186). Furthermore, the incidence of complications in the old-donor group did not differ according to the number of graft loss–related risk factors identified in this study (Supplemental Digital Content Figure S14, available at: http://links.lww.com/JS9/F186; Supplemental Digital Content 2, available at: http://links.lww.com/JS9/F186).

### Donor complications within 6 months postoperatively

Table 3 summarizes living donor complications within 6 months after donor hepatectomy. Grade III complication rates were similar between the old and young donors (2.6% vs. 3.8%, *P* = 0.672). There were no significant differences in the incidence of infectious, bleeding, vascular, or bile duct complications between the two groups.

**Table 3:** Living donor complications within 6 months after surgery

Variables	Old-donor (*n* = 374)	Young-donor (*n* = 3661)	*P*
Complication grade			0.672
No complication	355 (94.9)	3440 (94.0)	
Grade I~II	9 (2.4)	84 (2.3)	
Grade IIIA	8 (2.1)	94 (2.6)	
Grade IIIB	2 (0.5)	43 (1.2)	
Types of complications			
Wound complication	2 (0.5)	49 (1.3)	0.279
Intra-abdominal fluid collection	2 (0.5)	39 (1.1)	0.482
Intra-abdominal bleeding	2 (0.5)	21 (0.6)	1.000
Hepatic vein complication	0	2 (0.1)	1.000
Portal vein complication	0	16 (0.4)	0.396
Hepatic artery complication	0	0	
Bile duct complication	9 (2.4)	67 (1.8)	0.561
GI complications	1 (0.3)	6 (0.2)	1.000
Intestinal obstruction	0	2 (0.1)	1.000
Secondary peritonitis	0	1	1.000
Respiratory complication	3 (0.8)	9 (0.2)	0.167
Intubation-related complication	0	2 (0.1)	1.000
Vascular catheter-related complication	0	1	1.000
Psychiatric complication	0	0	
Other complication	2 (0.5)	16 (0.4)	1.000

## Discussion

In this Korean multicenter LDLT cohort, we found that increasing donor age to >50 years significantly reduces graft survival. Three key risk factors for graft loss in old-donor LDLT were identified: CIT ≥ 150 min, MELD score ≥ 20, and recipient BMI ≥ 25 kg/m^2^. When two or more of these factors were present, old-donor LDLT was significantly associated with poorer outcomes compared to young-donor LDLT, particularly within the first 6 postoperative months. This study uniquely demonstrates outcomes of old living donor LDLT in a multicenter cohort and highlights the relative feasibility of using old versus young donors, especially when DDLT is not available and old donors are the only option.

In LDLT, where partial graft regeneration is crucial postoperatively, aged liver grafts pose greater challenges compared to whole liver grafts in deceased donor LT (DDLT). Two large multicenter studies – the United Network for Organ Sharing database^[6]^ and the Japanese Liver Transplantation Society registry^[8]^ – identified living donors aged >50 years as an independent risk factor for patient survival in adult LDLT. However, these studies did not explore key factors specific to old-donor LDLT, limiting clinical applicability. A major strength of our study is its interaction analysis within a large LDLT cohort, providing practical insights for performing LDLT with old donors amid donor shortages.

A previous UNOS-based study demonstrated that D-MELD (donor age × MELD) effectively predicts post-LT survival^[20]^. A Japanese single-center study also reported its prognostic value in LDLT^[21]^. However, the application of D-MELD to LDLT is limited due to the younger age of living donors and differing implications of graft volume and steatosis compared to DDLT. In settings where an old living donor is the only option due to donor shortage, other clinical factors may be more critical. Based on our findings, we propose that accumulating risk factors – high MELD scores, recipient obesity, and prolonged CIT – may exceed the regenerative capacity of old liver grafts, impairing recovery, regeneration, and proper functioning^[22]^. In contrast, younger grafts may have greater regenerative potential to withstand hepatic injury.

The weakness of old liver grafts in the LT partly stems from their increased susceptibility to ischemia-reperfusion injury (IRI) compared to younger livers. Although ischemic time in LDLT is shorter than in DDLT, IRI remains a concern for partial old grafts due to their critical need for regenerative capacity for functional restoration. Old grafts exhibit impaired intracellular energy metabolism due to mitochondrial dysfunction, altered autophagy, and increased inflammatory response, exacerbating IRI severity^[23]^. In our study, graft loss in old-donor LDLT with two or more risk factors was most pronounced within the first 6 months post-LT, followed by a steady decline. While the incidence of early-stage complications, including biliary, vascular, bleeding issues, and acute rejections, was unaffected by the number of risk factors in old-donor LDLT, the presence of multiple risk factors likely worsens IRI-related and complication-induced liver damage, hindering graft regeneration and ultimately leading to poorer graft survival.

Our study found that old-donor LDLT poses a higher risk of graft loss, specifically in obese recipients. While higher BMI was found to be a protective factor in the overall Cox regression analysis, recipient obesity combined with an aged liver graft could impair graft function through various mechanisms. Excess adipose tissue in obese patients leads to increased leptin and decreased adiponectin, triggering heightened activation of immune cells and pro-inflammatory cytokines such as tumor necrosis factor-α and interleukin-6^[24]^. This pro-inflammatory state may exacerbate the inflammatory response to IRI in aged livers^[23]^. Early postoperative injury can further disrupt homeostatic inflammation in the liver, jeopardizing old liver grafts to chronic inflammation and inadequate function^[25]^. Metabolically, obesity reduces hepatic insulin sensitivity, hepatic glucose uptake, and increases fatty acid influx into the liver^[24]^, further compounding the limited gluconeogenesis and decline in mitochondrial function in aged livers, delaying regeneration after acute injuries^[3,22]^. As recipient BMI at the time of surgery may be influenced by factors such as ascites or edema, analyses using lean body weight or adjustments for fluid retention could offer more accurate assessments. However, such data were not available in this registry, which represents a limitation of the present study.

In our analysis, the risk of graft loss in old-donor LDLT increased significantly with MELD scores ≥ 20. Similarly, Ikegami *et al* reported that a D-MELD (donor age × MELD score) ≥900 predicted higher rates of graft dysfunction, sepsis, and mortality in LDLT^[21]^. For donors over 50 years, a MELD score >18 posed a significant risk of graft loss, aligning with our results. Recipients with high MELD scores require prompt graft recovery to meet elevated metabolic demands, but face greater risks of bleeding, transfusion requirements, renal dysfunction, and prolonged intensive care unit stays^[26]^, heightening the risk of life-threatening infection^[27–29]^. As the liver plays a key role in modulating systemic immune responses in severe infections like sepsis^[25,30]^, its functional restoration is particularly important in recipients with high MELD scores. The decreased regenerative capacity of old liver grafts may impair recovery from severe infections, further exacerbated by hepatic dysfunction, including hypoxic hepatitis and sepsis-induced cholestasis^[30]^. When deciding between LDLT with an old living donor or awaiting DDLT for high-MELD recipients, additional risk factors should be carefully assessed, as the benefits of LDLT over DDLT may diminish.

In our cohort, a CIT of 150 min was identified as the threshold beyond which the risk of old graft loss increases. Among the three risk factors analyzed, CIT is the only modifiable and preoperatively predictable variable. It is often influenced by donor anatomical complexity – such as multiple hepatic arteries, portal veins, bile ducts, or complex hepatic veins – which can prolong bench work and anastomosis time. Therefore, in cases involving old donors with anatomical complexity, careful planning to minimize CIT is essential. When combined with other risk factors, the cumulative risk must be fully recognized. A limitation of this study is the lack of donor anatomical data in the KOTRY registry, underscoring the need for future studies to evaluate the impact of anatomical complexity in old donor LDLT.

Although this study included a substantial number of LDLT cases, it has certain limitations. First, as only 5.1% of grafts in the old-donor LDLT group were non-right lobe grafts, the impact of graft type on safety could not be assessed. Second, although portal flow modulation is a common procedure for small grafts in Korea, the lack of detailed information on this practice in the KOTRY database prevented us from drawing definitive conclusions regarding the risk of low GRWR in old grafts. GRWR < 0.8 was not associated with increased graft loss in old-donor LDLT, likely due to the mitigating effects of portal flow modulation. Although the hazard might increase significantly with GRWR < 0.7^[8]^, the small sample size in our cohort (only 17 patients with GRWR < 0.7 in the old-donor group) restricted further analysis. The limitations regarding graft type and size should be further validated in study cohorts from other countries. Analysis of living liver donors aged over 60 years was not feasible due to the small number of such cases in the KOTRY registry, which also represents a limitation of this study. Finally, with only 14.7% (*n* = 55) of old-donor grafts having >10% steatosis in this study cohort, outcomes with higher steatosis thresholds (e.g., >20% or 30%) could not be evaluated. This limitation reflects current protocols at most centers, where living donors suspected of having fatty liver based on pretransplant controlled attenuation parameter or magnetic resonance imaging are advised to achieve a certain degree of weight loss before donation^[31]^.

## Conclusions

Graft survival in LDLT with old donors is significantly compromised by the presence of multiple risk factors (CIT ≥ 150 min, MELD score ≥ 20, and recipient BMI ≥ 25 kg/m^2^), although acceptable outcomes can still be achieved when one or no risk factors are present. Donor age was not associated with an increased risk of short-term donor complications. These findings underscore the importance of careful donor-recipient matching and risk factor minimization to ensure the viability of elderly donor grafts and to support the safe expansion of the living donor pool in LDLT.

## Supplementary Material

## Data Availability

All data used for analysis in this study are available from the corresponding author upon reasonable request.

## Research registration unique identifying number (UIN)

Name of the registry: CRIS (Clinical Research Information Service). Unique Identifying number or registration ID: KCT0010510. https://cris.nih.go.kr/cris/member/my/myCris.do.
