# Supplementary material for: Navigating the risk of living donor liver transplantation from older donors: analysis of 4035 cases from a multicenter cohort
Source: Int J Surg. 2025 Sep 22;112(1):1211–8. doi: 10.1097/JS9.0000000000003522 (PMC12825590; doi:10.1097/JS9.0000000000003522)
Supplement: Supplementary file 1 [file js9-112-1211-001.docx]

**Table of contents**

Supplementary Figure 12

Supplementary Figure 23

Supplementary Figure 34

Supplementary Figure 45

Supplementary Figure 55

Supplementary Figure 66

Supplementary Figure 76

Supplementary Figure 87

Supplementary Figure 97

Supplementary Figure 108

Supplementary Figure 118

Supplementary Figure 129

Supplementary Figure 139

Supplementary Figure 1410

Supplementary Table 111

Supplementary Table 212

Supplementary Table 213

**Figure S1. Overview of the study population**

LDLT, living donor liver transplantation; KOTRY, Korean organ transplantation registry**
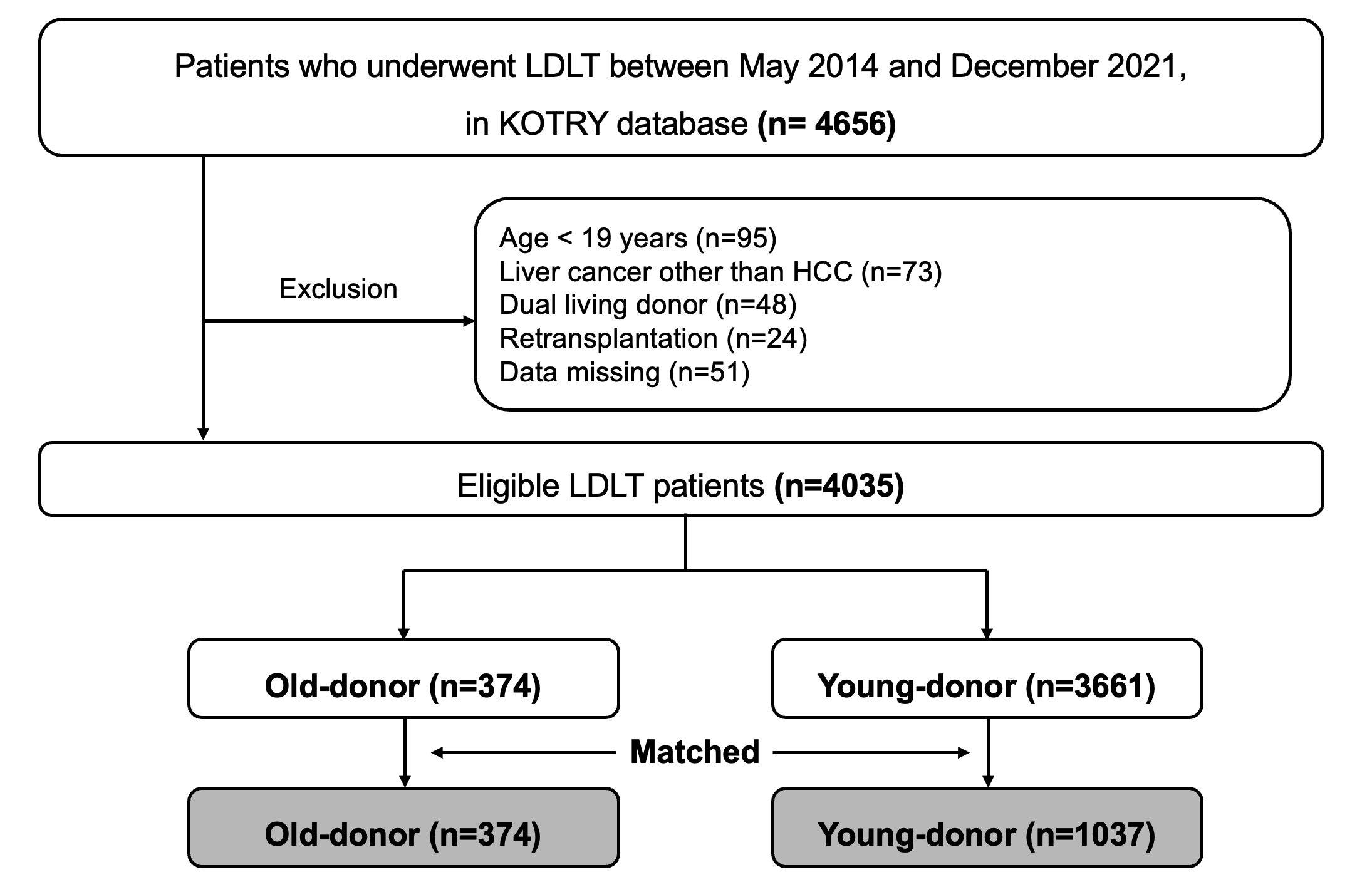
**

**Figure S2. Distribution and categorization of living donor age.** (A) Distribution of living donor age in the entire cohort. (B) Recipients are categorized by the cut-off of living donor age (50 years), which was determined on the smoothing spline curve for the risk of graft loss.


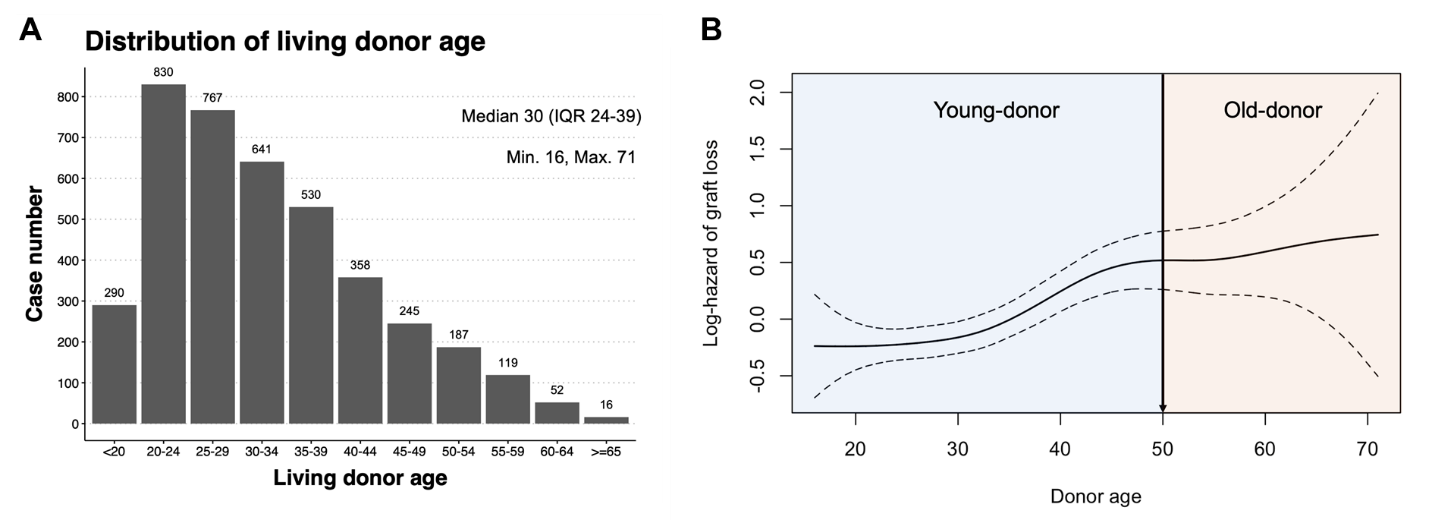


**Figure S3. Graft survival stratified by 10 year-intervals of living donor age**

LDLT, living donor liver transplantation

**
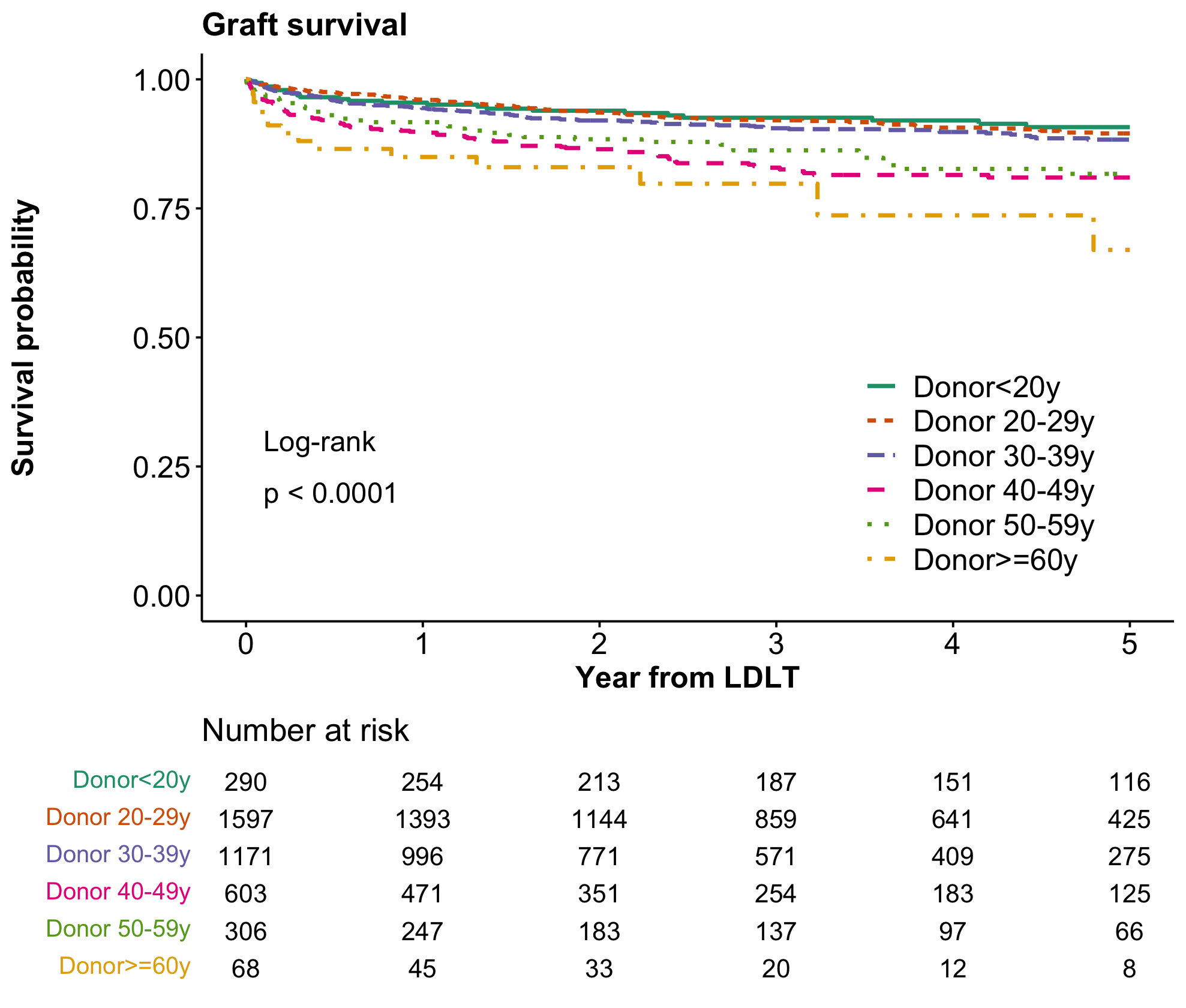
**

**Figure S4. Adjusted hazard for graft loss according to recipient age**


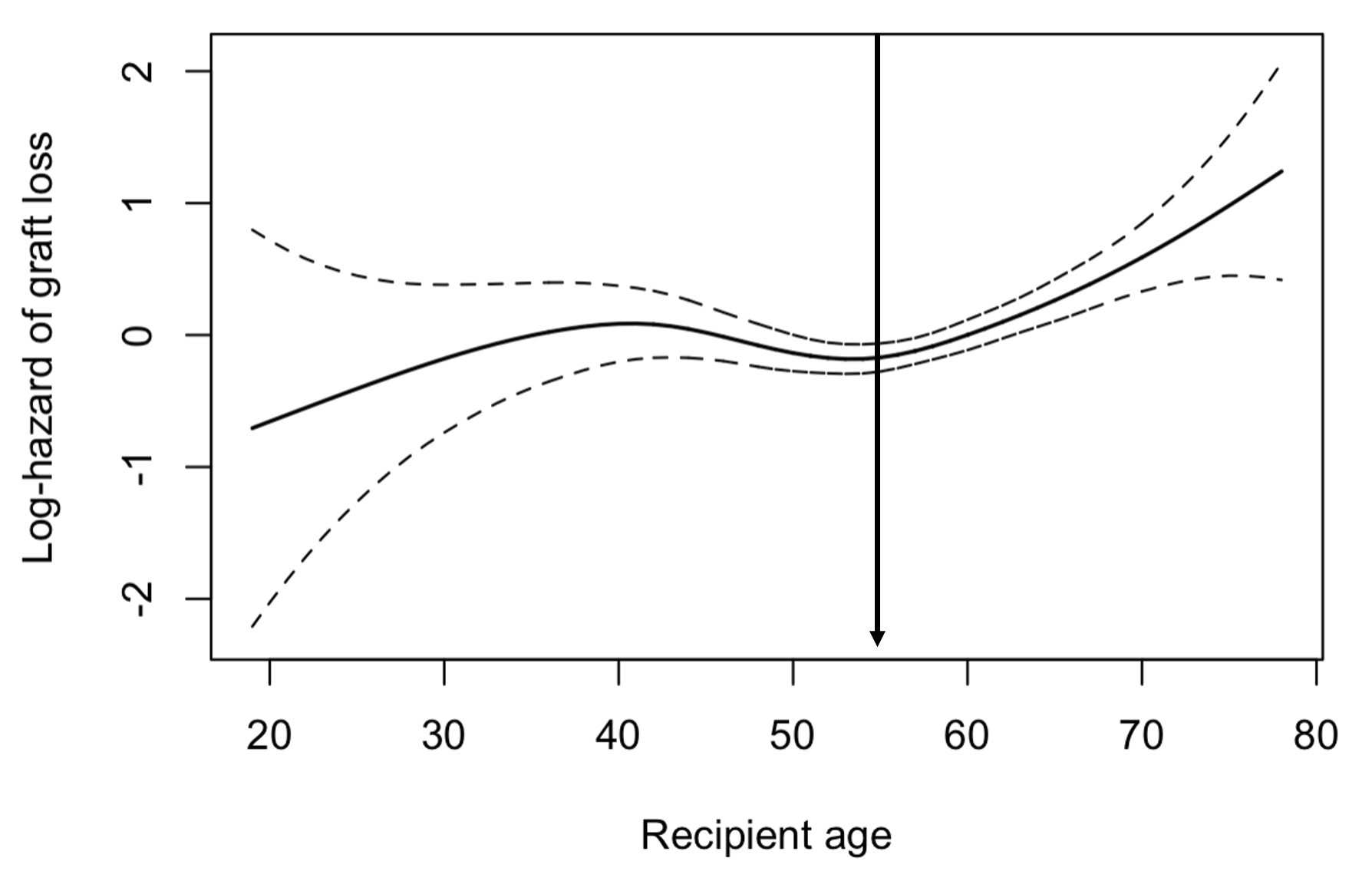


**Figure S5. Unadjusted hazard of Old-donor vs. Young-donor on LDLT survival according to recipient BMI**

LDLT, living donor liver transplantation; BMI, body mass index; HR, hazard ratio


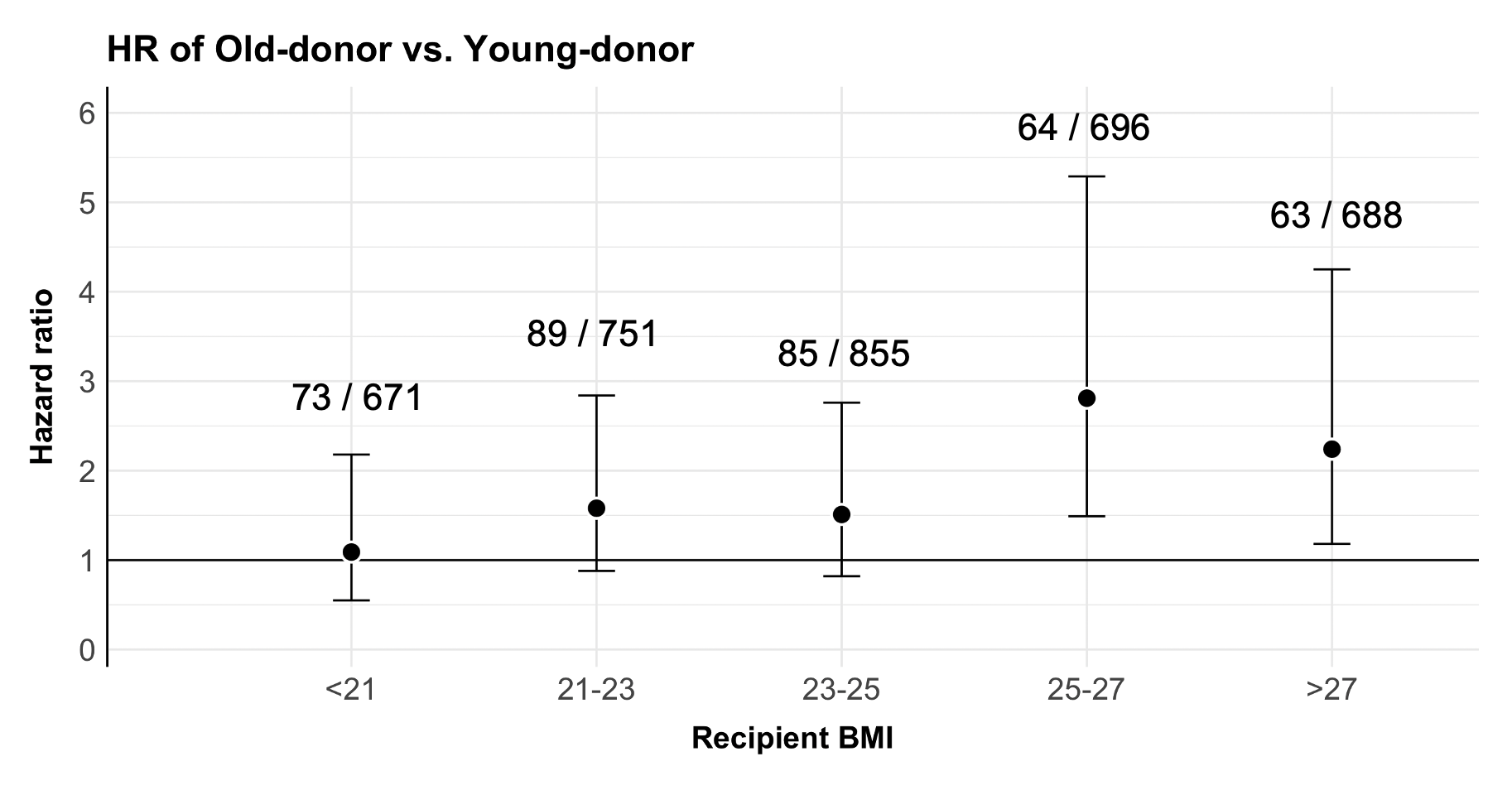


**Figure S6. Unadjusted hazard of Old-donor vs. Young-donor on LDLT survival according to MELD**

LDLT, living donor liver transplantation; MELD, model for end stage liver disease; HR, hazard ratio


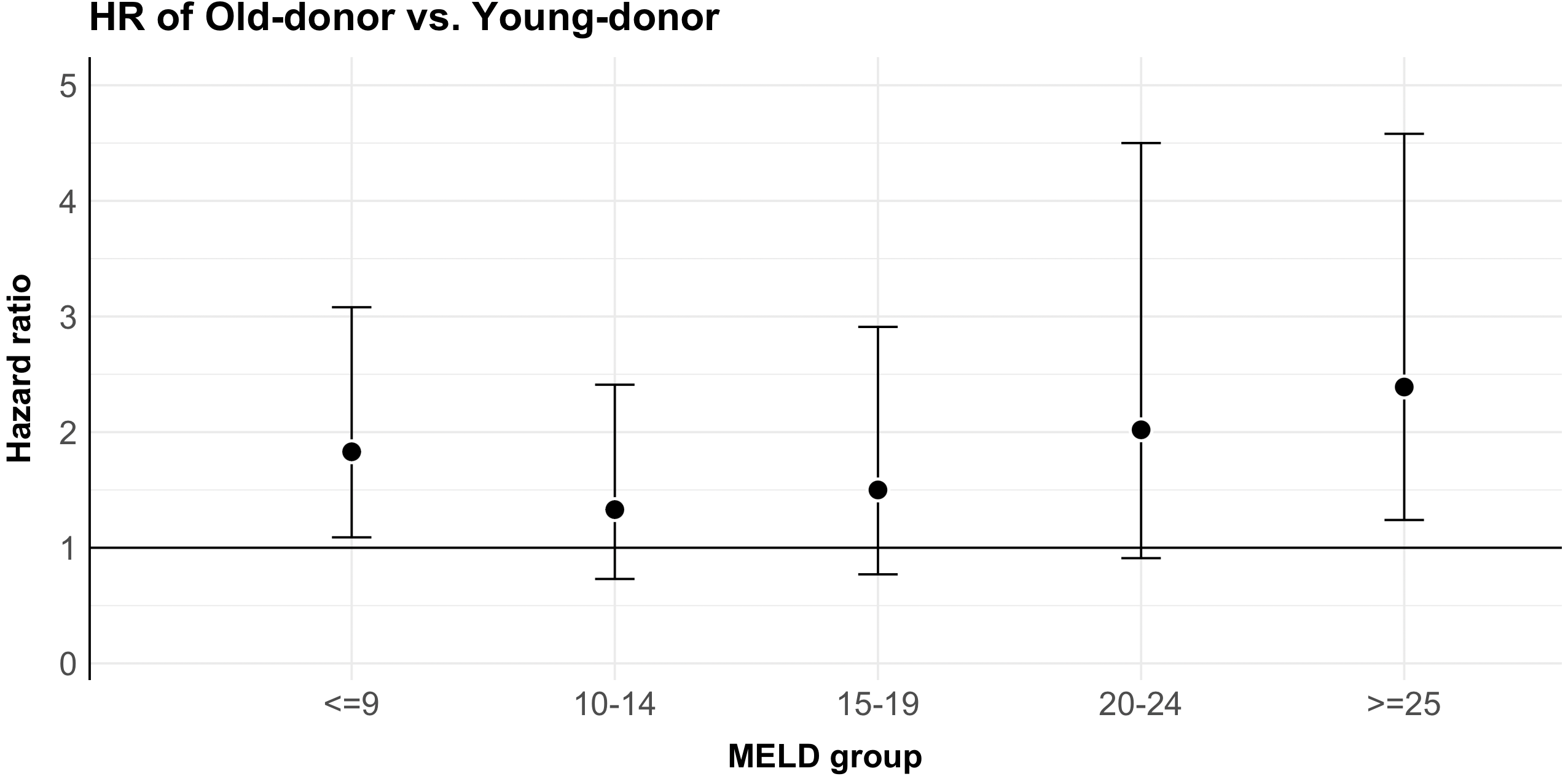


**Figure S7. Unadjusted hazard of Old-donor vs. Young-donor on LDLT survival according to CIT**

CIT, cold ischemic time; HR, hazard ratio


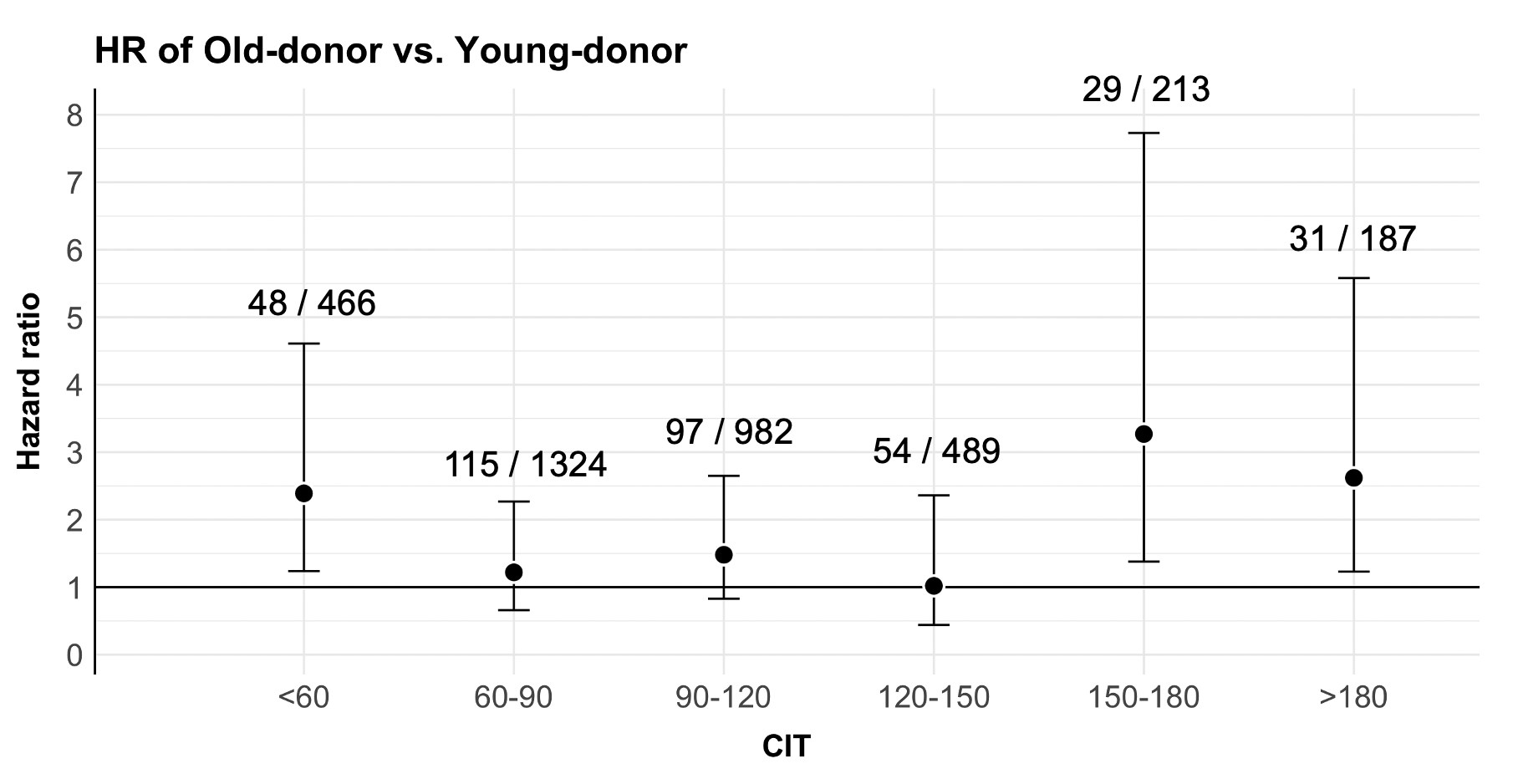


**Figure S8. Unadjusted hazard of Old-donor vs. Young-donor on LDLT survival according to donor BMI**

BMI, body mass index; HR, hazard ratio


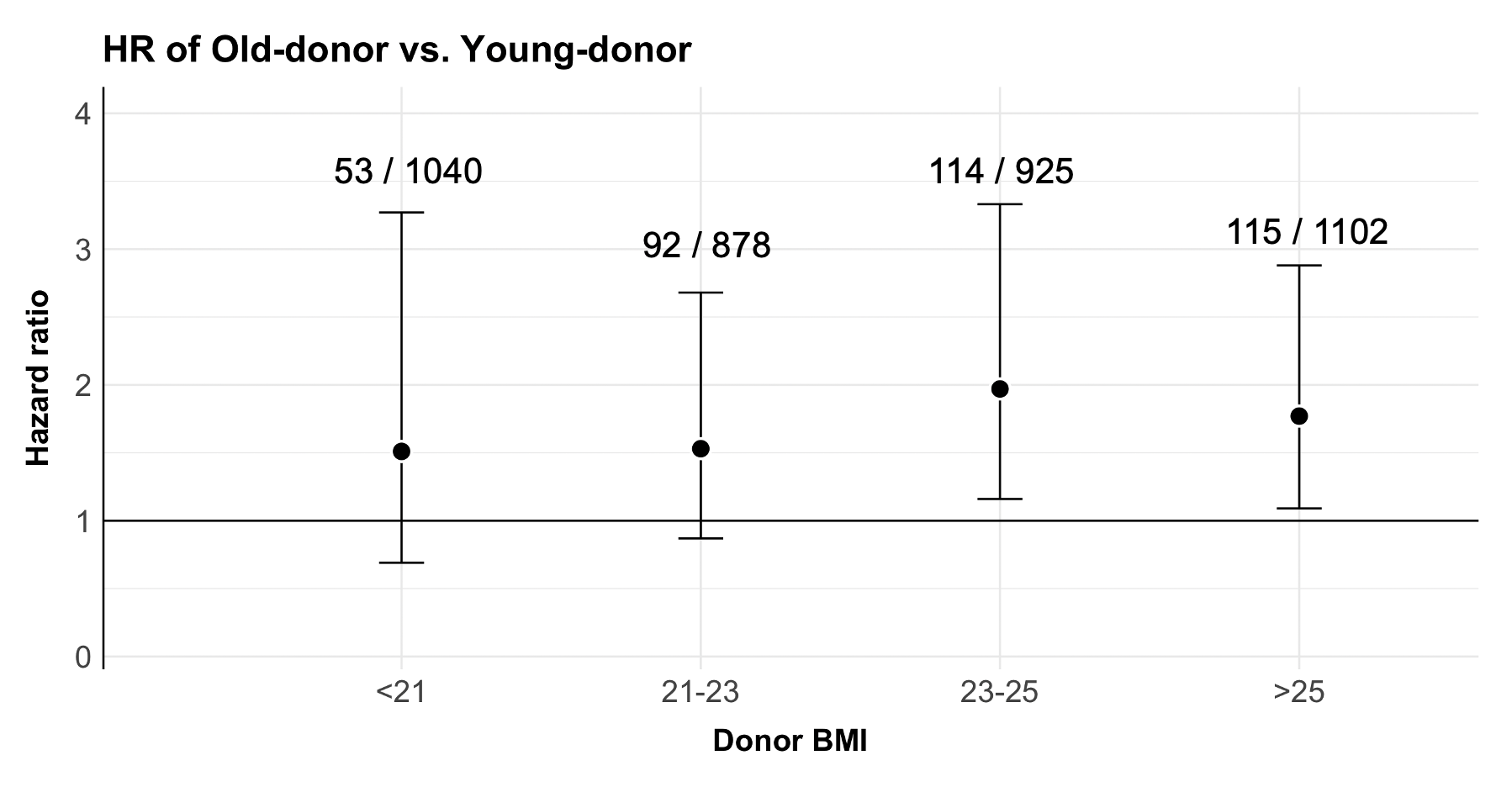


**Figure S9. Graft survival by GRWR and living donor age**

GRWR, graft-to-recipient weight ratio; LT, liver transplantation

**
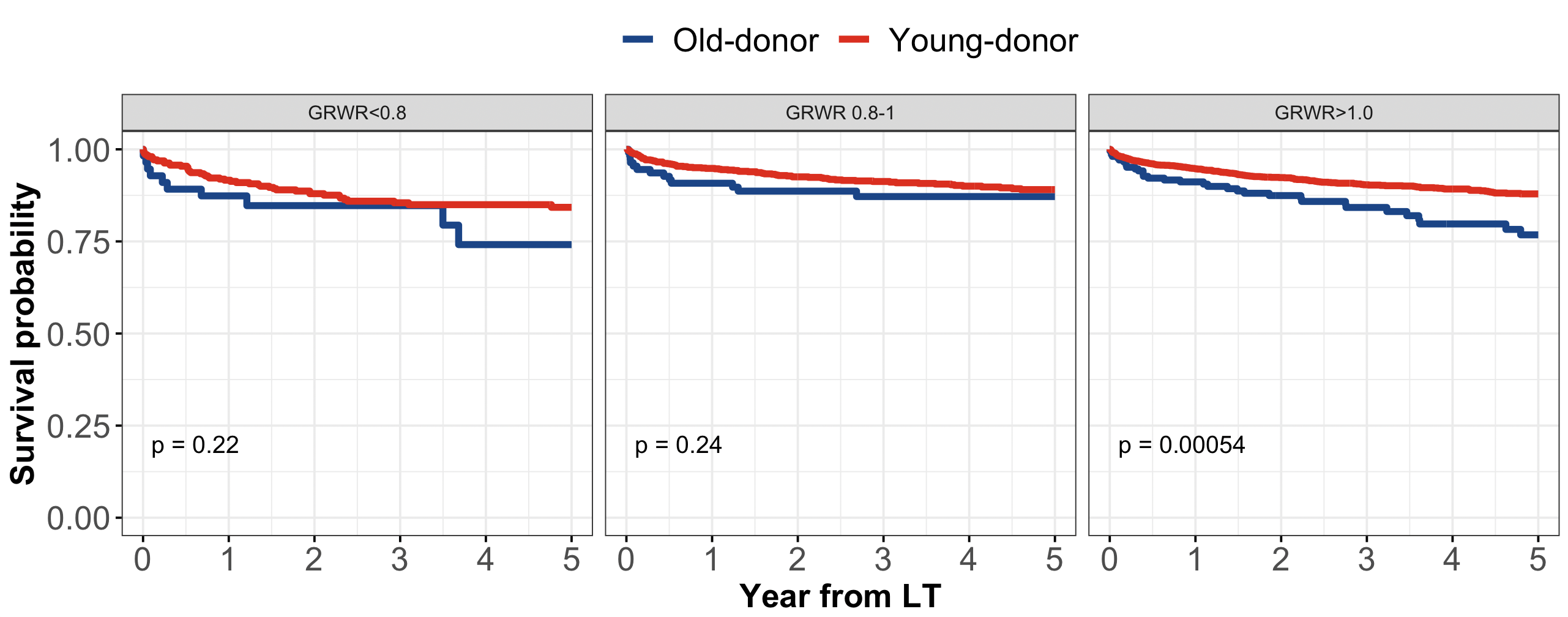
**

**Figure S10. Unadjusted hazard of Old-donor vs. Young-donor on LDLT survival according to GRWR**

LDLT, living donor liver transplantation; GRWR, graft-to-recipient weight ratio; HR, hazard ratio

**
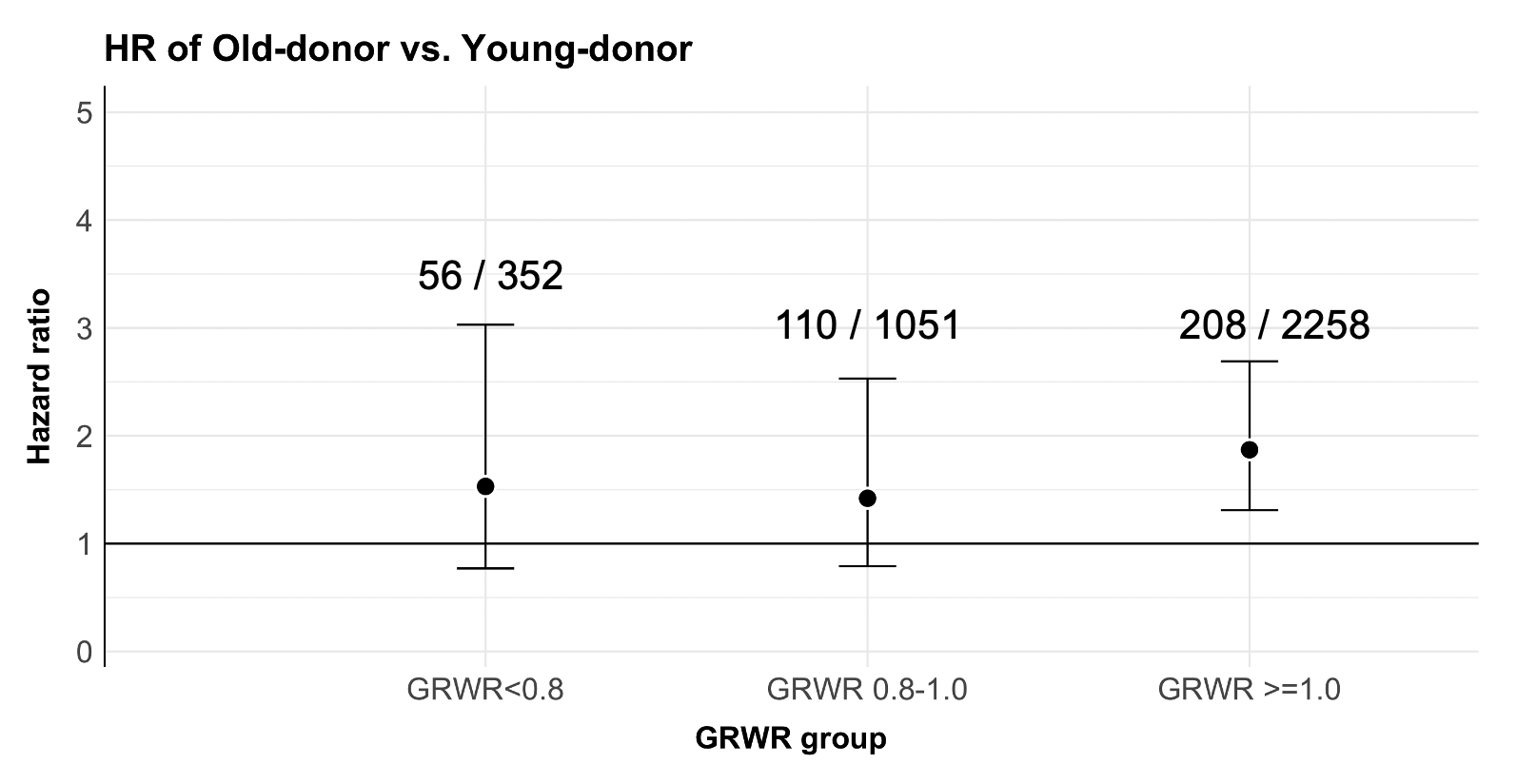
**

**Figure S11. Graft survival by graft steatosis and living donor age**

LT, liver transplantation

**
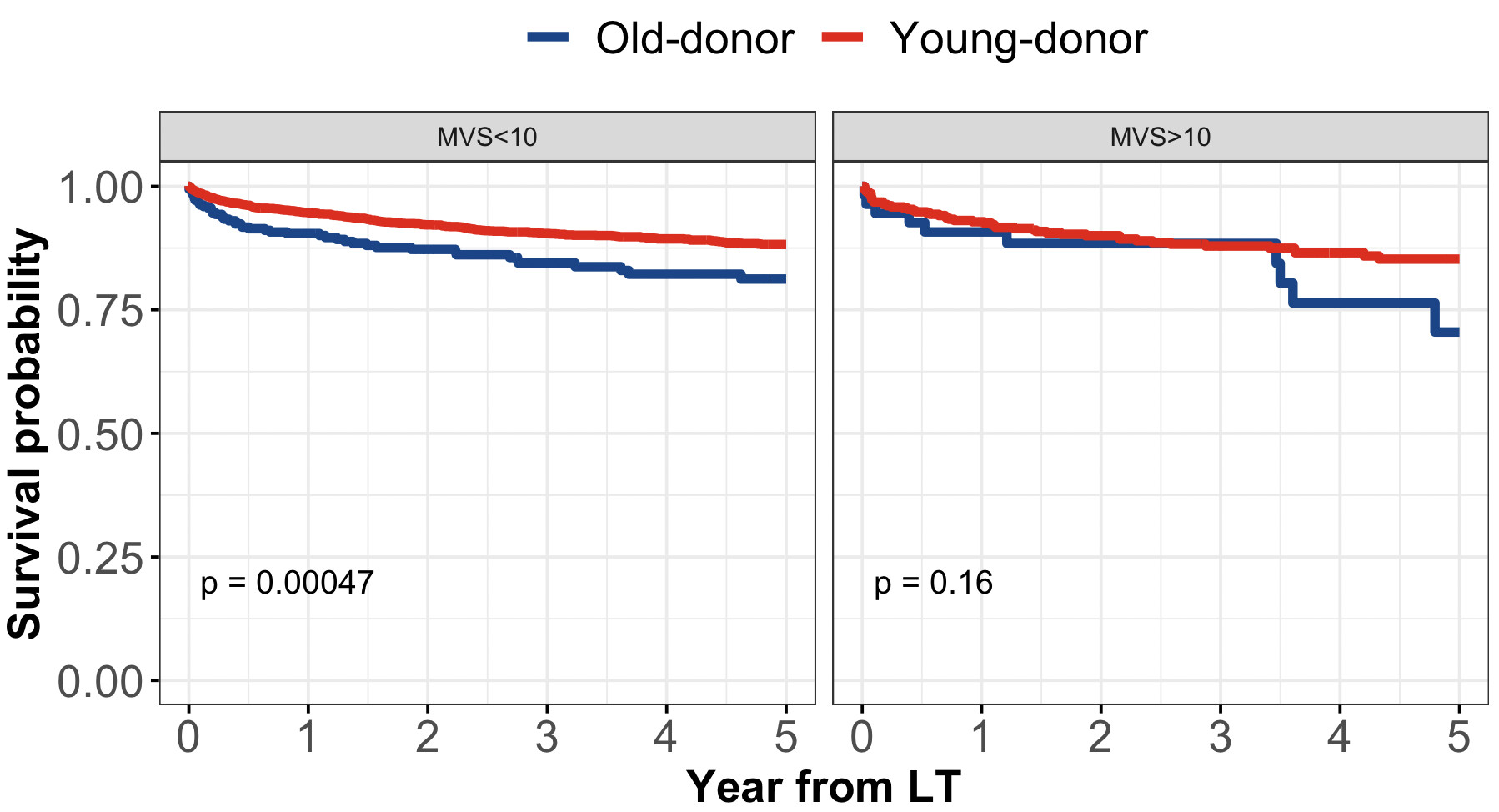
**

**Figure S12. Comparison of survival according to the number of risk factors for old-donor LDLT**

LDLT, living donor liver transplantation

**Figure S13. Graft survival stratified with donor age group and the number of risk factors**

LT, liver transplantation


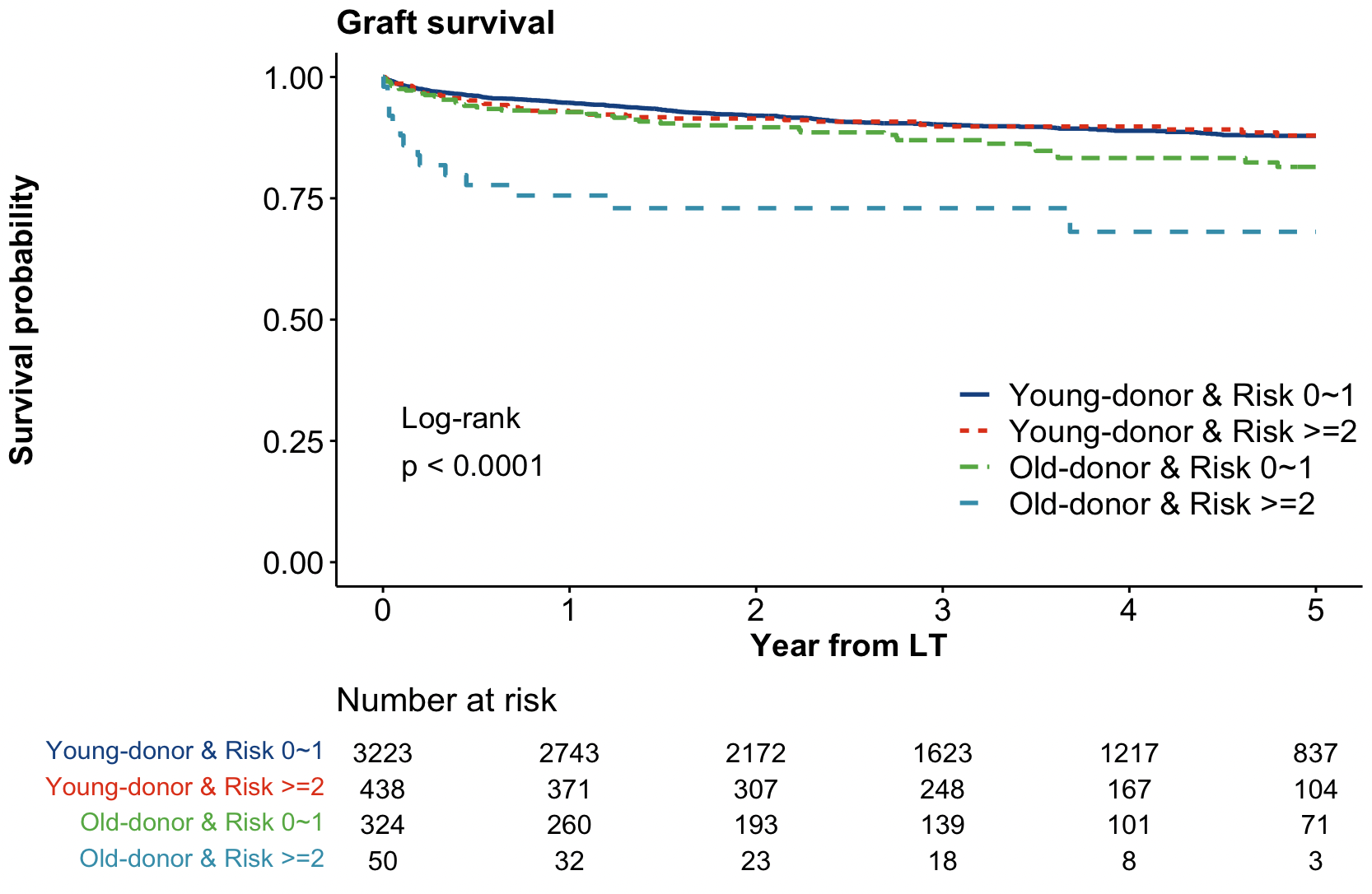


**Figure S14. Complications according to number of risk factors in old-donor LDLT**


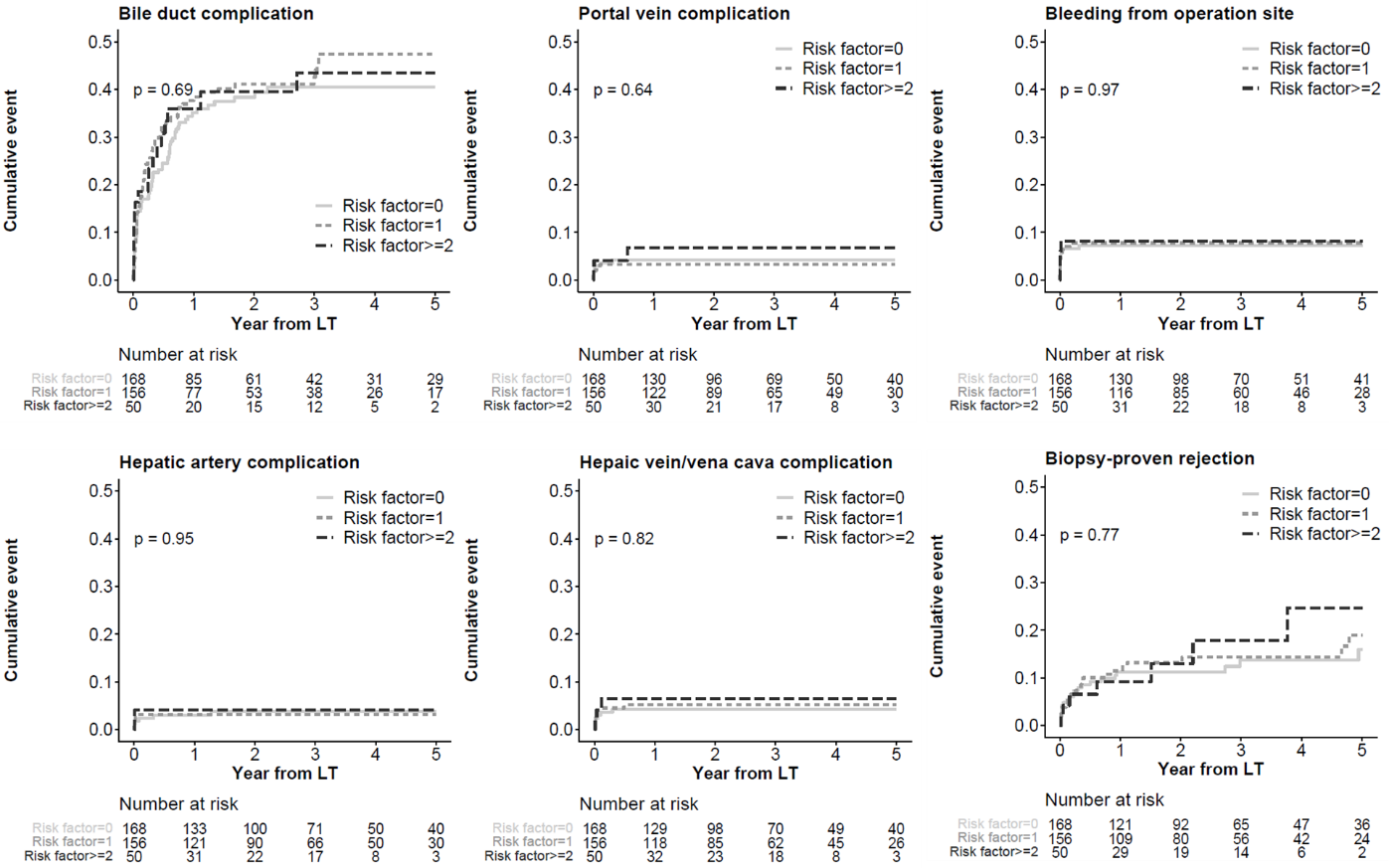


**Table S1. Multivariable Cox regression for graft survival**

| **Variables** |  | **HR (univariable)** | **HR (multivariable)** |
| --- | --- | --- | --- |
| Donor age group | Young-donor | **–** | **–** |
|  | Old-donor | 1.72 (1.30**–**2.27, p < 0.001) | 1.56 (1.17**–**2.07, p = 0.002) |
| Age |  | 1.02 (1.01**–**1.03, p = 0.003) | 1.01 (1.00**–**1.03, p = 0.026) |
| Sex | Male | 1.00 (0.81**–**1.24, p = 0.993) | **–** |
| Year of LT | 2018-2021 | 1.36 (1.10**–**1.66, p = 0.004) | 1.23 (0.99**–**1.52, p = 0.056) |
| BMI |  | 0.97 (0.95**–**1.00, p = 0.051) | 0.96 (0.93**–**0.99, p = 0.004) |
| Underlying group | Viral | **–** | **–** |
|  | Alcoholic | 1.17 (0.93**–**1.47, p = 0.175) | 1.30 (1.00**–**1.69, p = 0.047) |
|  | Others | 1.30 (1.00**–**1.71, p = 0.054) | 1.53 (1.14**–**2.05, p = 0.005) |
| Diabetes mellitus | Yes | 1.23 (1.00**–**1.51, p = 0.050) | 1.16 (0.94**–**1.44, p = 0.168) |
| HCC | Yes | 1.29 (1.06**–**1.58, p = 0.010) | 2.02 (1.57**–**2.60, p < 0.001) |
| Refractory ascites | Yes | 1.70 (1.38**–**2.10, p < 0.001) | 1.53 (1.21**–**1.93, p < 0.001) |
| Encephalopathy | Yes | 1.87 (1.43**–**2.43, p < 0.001) | 1.46 (1.08**–**1.96, p = 0.014) |
| Hospitalization | OPD | **–** | **–** |
|  | Ward | 0.82 (0.67**–**1.00, p = 0.047) | 0.92 (0.75**–**1.14, p = 0.458) |
|  | ICU | 2.29 (1.55**–**3.39, p < 0.001) | 1.85 (1.18**–**2.90, p = 0.008) |
| MELD |  | 1.02 (1.01**–**1.03, p < 0.001) | 1.02 (1.01**–**1.04, p = 0.008) |
| ABO incompatibility | Yes | 1.55 (1.26**–**1.91, p < 0.001) | 1.61 (1.30**–**1.99, p < 0.001) |
| Donor sex | Male | 0.85 (0.70**–**1.04, p = 0.112) | **–** |
| Donor BMI |  | 1.00 (0.97**–**1.03, p = 0.947) | **–** |
| GRWR | GRWR<0.8 | 1.49 (1.13**–**1.96, p = 0.005) | 1.66 (1.23**–**2.25, p = 0.001) |
| Graft type group | Other than right | 1.88 (1.33**–**2.67, p < 0.001) | 1.55 (1.07**–**2.24, p = 0.021) |
| Graft steatosis | ≥10% | 1.31 (0.99**–**1.72, p = 0.056) | 1.18 (0.90**–**1.56, p = 0.234) |
| Cold ischemic time |  | 1.25 (1.12**–**1.40, p < 0.001) | 1.23 (1.09**–**1.38, p = 0.001) |

HR, hazard ratio; LT, liver transplantation; BMI, body mass index; OPD, outpatient department; ICU, intensive care unit; MELD, model for end stage liver disease; GRWR, graft-to-recipient weight ratio

**Table S2. Cause of graft loss**

|  | **Old-donor**  (N=57) | **Young-donor**  (N=357) | **p** |
| --- | --- | --- | --- |
| **Cause of graft loss** |  |  | 0.095 |
| -Death with functioning graft | 33 (57.9) | 250 (70.0) |  |
| -Primary nonfunction | 8 (14.0) | 20 (5.6) |  |
| -Hepatic artery complication | 3 (5.3) | 11 (3.1) |  |
| -Hepatic vein complication | 1 (1.8) | 2 (0.6) |  |
| -Portal vein complication | 1 (1.8) | 2 (0.6) |  |
| -Biliary complication | 2 (3.5) | 8 (2.2) |  |
| -Rejection | 1 (1.8) | 12 (3.4) |  |
| -Drug toxicity | 1 (1.8) | 0 (0.0) |  |
| -Recurrent liver disease | 0 (0.0) | 4 (1.1) |  |
| -Recurrent HCC | 3 (5.3) | 28 (7.8) |  |
| -Others | 2 (3.5) | 7 (2.0) |  |
| -Unknown | 2 (3.5) | 13 (3.6) |  |

**Table S3. Principles for categorizing continuous variables for subgroup analyses.**

| **Variable (cutoff for subgroups)** | **Principles** |
| --- | --- |
| Recipient age (55 years) | Hazard for graft loss starts to increase from age 55 years (referenced to Figure S4) |
| Recipient BMI (25 kg/m^2^) | Hazard of Old-donor became significant at a recipient BMI over 25 (referenced to Figure S5) |
| MELD (20) | Hazard of Old-donor tended to be significant over MELD 20, although the result was marginal between 20~24, possible due to the small sample size (P = 0.086). (referenced to Figure S6). |
| Donor BMI (23 kg/m^2^) | Hazard of Old-donor turned to be significant over donor BMI 23 (referenced to Figure S8) |
| GRWR (0.8) | Traditional cutoff for safe LDLT |
| CIT (150 min) | Hazard of Old-donor turned to be significant over CIT 150 (referenced to Figure S7) |

BMI, body mass index; MELD, model for end stage liver disease; GRWR, graft-to-recipient weight ratio; LDLT, living donor liver transplantation; CIT, cold ischemic time

**Table S4. Surgical complications in recipient**

| **Types of complication** | | **Cumulative incidence** | | | **Adjusted^†^** |  |
| --- | --- | --- | --- | --- | --- | --- |
|  |  | **1-yr** | **3-yr** | **5-yr** | **HR (95% CI)** | **P** |
| Surgical complications |  |  |  |  |  |  |
| Bile duct | **Young-donor** | 24.0% | 26.9% | 27.8% | Reference |  |
|  | **Old-donor** | 36.7% | 41.9% | 43.9% | 1.41 (1.18-1.68) | <0.001 |
| Hepatic artery | **Young-donor** | 2.8% | 2.9% | 2.9% | Reference |  |
|  | **Old-donor** | 3.3% | 3.6% | 3.6% | 1.08 (0.60-1.92) | 0.805 |
| Portal vein | **Young-donor** | 2.5% | 2.7% | 2.8% | Reference |  |
|  | **Old-donor** | 4.1% | 4.1% | 4.1% | 1.46 (0.84-2.51) | 0.178 |
| Hepatic vein | **Young-donor** | 4.4% | 4.5% | 4.7% | Reference |  |
|  | **Old-donor** | 4.9% | 4.9% | 4.9% | 0.96 (0.59-1.57) | 0.865 |
| Bleeding from operation site | **Young-donor** | 4.9% | 4.9% | 4.9% | Reference |  |
|  | **Old-donor** | 7.6% | 7.6% | 7.6% | 1.33 (0.89-1.99) | 0.167 |
| Biopsy-proven rejection | **Young-donor** | 5.6% | 6.8% | 7.5% | Reference |  |
|  | **Old-donor** | 11.1% | 14.4% | 18.4% | 1.63 (1.13-2.33) | 0.009 |

Adjusted Cox regression models were established including covariates of which P values were <0.10 in univariate analyses.

**Table S5. Bile duct complication and biopsy-proven rejection by donor age and graft loss**

|  | **Young-donor** | |  | **Old-donor** | |  |
| --- | --- | --- | --- | --- | --- | --- |
|  | Survive  (n=3304) | Graft loss  (n=357) | P | Survive  (n=317) | Graft loss  (n=57) | P |
| **Bile duct complication** |  |  | 0.301 |  |  | >0.999 |
| No | 2438 (73.8) | 273 (76.5) |  | 195 (61.5) | 35 (61.4) |  |
| Yes | 866 (26.2) | 84 (23.5) |  | 122 (38.5) | 22 (38.6) |  |
| **Biopsy-proven rejection** |  |  | 0.008 |  |  | >0.999 |
| No | 3098 (93.8) | 321 (89.9) |  | 275 (86.8) | 49 (86.0) |  |
| Yes | 206 (6.2) | 36 (10.1) |  | 42 (13.2) | 8 (14.0) |  |
