# Supplementary material for: Navigating the risk of living donor liver transplantation from older donors: analysis of 4035 cases from a multicenter cohort
Source: Int J Surg. 2025 Sep 22;112(1):1211–8. doi: 10.1097/JS9.0000000000003522 (PMC12825590; doi:10.1097/JS9.0000000000003522)
Supplement: Supplementary file 2 [file js9-112-1211-002.docx]

| **STROCSS 2025 Guideline Checklist** | | | |
| --- | --- | --- | --- |
| **Topic** | **Item** | **Item Description** | **Page Number** |
| **Title** | 1 | - The word ‘cohort’ or ‘cross-sectional’ or ‘case-control’ is included* - Temporal design of study is stated (e.g. retrospective or prospective) - The focus of the study is clearly stated (e.g. population, setting, disease, exposure/intervention, outcome etc.)   **STROCSS 2025 guidelines apply to all observational studies (e.g. cohort, cross-sectional, case-control etc.)* | 1 |
| **Highlights** | 2 | - Include three to five bullet points that summarise the key findings of the study - Provide a brief background to the study, the key results and clinical relevance | 1 |
| **Abstract** | 3a | - Provide a structured abstract that includes the following headings:  1. Background 2. Methods 3. Results 4. Conclusions | 1 |
|  | 3b | Background Briefly describe:   - Relevant context - Scientific rationale for this study - Aims and objectives | 1 |
|  | 3c | Methods Briefly describe:   - Type of study design (e.g. cohort, case-control, cross-sectional etc.) - Specification of study design (e.g. retro-/prospective, single/multi-centred etc.) - All patient groups involved, including control group, if applicable - Exposure/interventions (e.g. type, operators, recipients, dates and time frames etc.) - Outcome measures - explicitly state primary and secondary outcome(s), where appropriate - Statistical methods of assessment used, where applicable | 1 |
|  | 3d | Results  Briefly describe:   - Summary data - Principal findings with qualitative descriptions - Statistical findings and their significance, where appropriate | 1 |
|  | 3e | Conclusion   - Describe key conclusions briefly - Refer to implications for clinical practice and public health - Describe the need for and direction of future research - Include a concise statement that encapsulates the significance of the research and its contribution to the field | 1 |
| **Keywords** | 4 | - Include three to six keywords that identify what is covered in the study (e.g. patient population, diagnosis, or surgical intervention) - Include study type as a keyword (e.g. cohort study, cross-sectional study, case-control study etc.) - Include surgical speciality as one of the keywords. - Include study location as one of the keywords. | 1 |
| **Artificial Intelligence (AI)**  (some journals may prefer this in the methods and/or acknowledgments section and it should also be declared in the cover letter) | 5 | Declaration of whether any AI was used in the research and manuscript development   - If no, proceed to item 6. - If yes, proceed to item 5a. | No |
|  | 5a | **Purpose and Scope of AI Use**   - Precisely state why AI was employed (e.g. development of research questions, language drafting, statistical analysis/summarisation, image annotation, etc). - Was generative AI utilised and if so, how? - Clarify the stage(s) of the reporting workflow affected (planning, writing, revisions, figure creation).   Confirmation that the author(s) take responsibility for the integrity of the content affected/generated. |  |
|  | 5b | **AI Tool(s) and Configuration**   - Name each system (vendor, model, major version/date). - State the date it was used - Specify relevant parameters (e.g. prompt length, plug-ins, fine-tuning, temperature). - Declare whether the tool operated locally on-premises, or via a cloud API and any integrations with other systems. |  |
|  | 5c | **Data Inputs and Safeguards**   - Describe categories of data provided to the AI (patient text, de-identified images, literature abstracts). - Confirm that all inputs were de-identified and compliant with GDPR/HIPAA. - Note any institutional approvals or data-sharing agreements obtained. |  |
|  | 5d | **Human Oversight and Verification**   - Identify the supervising author(s) who reviewed every AI output. - Detail the process for fact-checking, clinical accuracy checks - State whether any AI-generated text/figures were edited or discarded. - Acknowledge the limitations of AI and its use |  |
|  | 5e | **Bias, Ethics and Regulatory Compliance**   - Outline steps taken to detect and mitigate algorithmic bias (e.g. cross-checking against under-represented populations). - Affirm adherence to relevant ethical frameworks.   Disclose any conflicts of interest or financial ties to AI vendors. |  |
|  | 5f | **Reproducibility and Transparency**   - Provide the exact prompts or code snippets (as supplementary material if lengthy). - Supply version-controlled logs or model cards where possible.   If applicable, state repository, hyperlink or digital object identifier (DOI) where AI-generated artefacts can be accessed, enabling attempts at independent replication of the query/input. |  |
| **Introduction** | 6a | **Introduction**  By referencing key literature throughout, comprehensively describe:   - Relevant background and scientific rationale for study - Aims and objectives - Research question and hypotheses, where appropriate - Potential impact of research on future clinical practice - Economic relevance of study to society | 2 |
|  | 6b | **Guideline citation**   - At the end of the introduction, refer to the STROCSS 2025 publication by stating: ‘This cohort/cross-sectional/case-control study has been reported in line with the STROCSS guidelines [*include citation*]’ | 1 |
| **Methods:**  **Study Design** | 7a | **Study design**   - State the type of study design (e.g. cohort, cross-sectional, case-control etc.) - Describe other key elements of study design (e.g. retro-/prospective, single/multi-centred etc.) - Specify the duration of the study, including start and end dates | 2 |
|  | 7b | **Setting and timeframe of research**  Comprehensively describe:   - Specific geographical location - Nature of institution (e.g. primary/secondary/tertiary care setting, district general hospital/teaching hospital, public/private, low-resource setting etc.) - Timeline for study, including dates for recruitment, exposure, follow-up, data collection etc. - Any deviations from the initial study design plan or changes to the timeline during the research, with reasons and implications stated | 2 |
|  | 7c | **Study groups**   - Total number of participants - Number of groups - Number of participants in each group - Detail exposure/intervention allocated to each group - Inclusion and exclusion criteria with clear definitions | 2 |
|  | 7d | **Subgroup analysis**  Comprehensively describe:   - How subgroups were defined - Planned subgroup analyses - Methods used to examine subgroups and their interactions | 3 |
|  | 7e | **Follow up**  If applicable, comprehensively describe:   - Time, length, frequency, location and methods of follow-up (e.g. mail, telephone, with whom etc.) - Any specific long-term surveillance requirements (e.g. imaging surveillance of endovascular aneurysm repair) - Any specific post-operative instructions (e.g. post-operative medications, targeted physiotherapy etc.) | 2 |
| **Methods:**  **Participant Recruitment** | 8a | **Recruitment**  Comprehensively describe:   - Period of recruitment - Methods of recruitment to each patient group (e.g. all at once, in batches, continuously till desired sample size is reached etc.) - Sources of recruitment (e.g. physician referral, study website, social media, posters etc.) - Any monetary/non-monetary incentivisation of participants to encourage involvement should be declared *(the nature of any incentives provided must be clarified)* - Any challenges encountered during the recruitment processes, including how they were addressed | **2** |
|  | 8b | **Sample size**  Comprehensively describe:   - Analysis to determine optimal sample size for study accounting for population/effect size - Power calculations with justifications for chosen statistical power, where appropriate - Margin of error calculation - Any associated ethical considerations | 2 |
| **Methods:**  **Intervention and Outcomes** | 9a | **Pre-intervention considerations**  Comprehensively describe any preoperative patient optimisation:   - Lifestyle optimization (e.g. weight loss, smoking cessation, glycaemic control etc.) - Medical optimisation (e.g. medication review, treating hypothermia/-volemia/-tension, ICU care etc.) - Procedural optimisation (e.g. nil by mouth, enema etc.) - Other (e.g. psychological support, physiotherapy etc.) | 2 |
|  | 9b | **Intervention**  Comprehensively describe:   - Type of intervention and reasoning (e.g. pharmacological, surgical, physiotherapy, psychological etc.) - Aim of intervention (e.g. preventative/therapeutic) - Total cost of performing the intervention - Degree of novelty of intervention - Any learning required for intervention - Prevalence or frequency at which the intervention is performed - Concurrent treatments (e.g. antibiotics, analgesia, antiemetics, VTE prophylaxis etc.) - Manufacturer and model details, where appropriate | 2 |
|  | 9c | **Intra-intervention considerations**  Using figures and other media to illustrate wherever appropriate, comprehensively describe:   - Details pertaining to administration of intervention (e.g. anaesthetic, positioning, location, preparation, equipment needed, devices, sutures, operative techniques, operative time etc.) - For pharmacological therapies, the formulation, dosages, routes, strength and durations - For surgery, any post-operative instruction (e.g. when to remove staples or sutures) - The degree of novelty for a surgical technique/device (e.g. ‘first in human’) | 2 |
|  | 9d | **Operator details**  Comprehensively describe:   - Requirement for additional training - Learning curve for technique, including how it was evaluated (e.g. number of cases required to reach a defined level of proficiency) - Relevant training, specialisation and operator’s experience (e.g. average number of the relevant procedures performed annually) - Any institutional support that was provided to operators to facilitate their training | 2 |
|  | 9e | **Setting of intervention**  Comprehensively describe:   - Setting in which the intervention was performed - Level of experience the centre has in performing the intervention | 2 |
|  | 9f | **Quality control**  Comprehensively describe:   - Measures taken to reduce inter-operator variability (e.g. regular team meetings, calibration exercises) - Measures taken to ensure consistency in other aspects of intervention delivery (e.g. data collection) - Measures taken to ensure quality in intervention delivery | 2 |
|  | 9g | **Post-intervention considerations**  Comprehensively describe:   - Post-operative instructions and care (e.g. avoid heavy lifting, dietary restrictions etc.) - Follow-up measures - Future surveillance requirements (e.g. blood tests, imaging etc.) - How patient engagement with post-intervention instructions will be encouraged and monitored - If applicable, the criteria for patient discharge from the medical facility | 3 |
|  | 9h | **Definition of outcomes**   - Define primary outcomes, including validation with full reference to relevant studies, where applicable - Define secondary outcomes, where appropriate - Describe methods or instruments used to measure each outcome, with full reference given if validated - Describe follow-up period for outcome assessment, divided by group | 3 |
|  | 9i | **Statistics**  Comprehensively describe:   - Statistical tests and statistical package(s)/software used - Rationale behind the statistical tests/software of choice - Confounders and their control, if known - Analysis approach (e.g. intention to treat/per protocol) - Any subgroup analyses - Level of statistical significance - How the results of the statistical analyses are presented (e.g. p-values, confidence intervals, point estimates etc.) | 4 |
| **Results** | 10a | **Participants**  Comprehensively describe:   - With reasons, the flow of participants (recruitment, non-participation, cross-over and withdrawal), using a figure to illustrate where appropriate - Population demographics (e.g. age, gender, relevant socio-economic features, prognostic features etc.) - Any significant numerical differences across groups - If applicable, the longitudinal changes in participant flow/demographics over time | 4 |
|  | 10b | **Participant comparison**   - Include table comparing baseline characteristics of cohort groups, with statistical data included - In a concise manner, highlight the principal, significant findings - Describe any group matching, with methods | 5 |
|  | 10c | **Outcomes**  Comprehensively describe:   - Clinician-assessed and patient-reported outcomes (e.g. questionnaires with quality-of-life scales) for each group - Expected versus attained outcomes, as assessed by the clinician* - Primary and secondary outcomes, as previously defined (8i) - Details of when the outcomes were recorded (e.g. at how many months/years post-operatively) - Relevant photographs and imaging are desirable - Any confounding factors and state which ones are adjusted and how - Any changes to interventions, with rationale and diagram, if appropriate   **NB: reference relevant literature to inform expected outcomes* | 5 |
|  | 10d | **Tolerance**  Comprehensively describe:   - Assessment of tolerability of exposure/intervention within patient groups - Methods of measuring tolerance/adherence - If applicable, specific patient perspectives - Whether these results will have an impact on the long-term applicability of the findings in clinical practice - Loss to follow-up (fraction and percentage), with reasons | 5 |
|  | 10e | **Complications**  Comprehensively describe:   - Adverse events, and classify according to Clavien-Dindo classification* - Timing of adverse events - Precautionary measures taken to prevent complications (e.g. antibiotic or venous thromboembolism prophylaxis) - Management of adverse events (e.g. blood transfusion, wound care, revision surgery etc.) - If applicable, whether the complication was reported to the national agency/pharmaceutical company - If applicable, specify whether any complications were discussed locally and the impact of such discussions (e.g. during team morbidity & mortality meetings) - State explicitly if there were no complications/adverse outcomes   **Dindo D, Demartines N, Clavien P-A. Classification of Surgical Complications. A New Proposal with Evaluation in a Cohort of 6336 Patients and Results of a Survey. Ann Surg. 2002; 240(2): 205-213* | 6 |
|  | 10f | **Key results**  Describe:   - Key findings, supported by relevant raw data and corresponding statistical analyses with significance | 6 |
| **Discussion** | 11a | **Principal findings**  By referencing key, relevant literature throughout, comprehensively describe:   - Summary of key findings and conclusions - Rationale behind conclusions drawn - Comparison to current gold standard of care, current guidelines or similar research - Implications of findings for future clinical practice and guidelines - Relevant hypothesis generation | 7 |
|  | 11b | **Strengths and limitations**  Comprehensively describe:   - Strengths of the study - Weaknesses and limitations of the study - Measures taken to overcome the limitations, if applicable - Potential impact on results and their interpretation - Assessment and management of bias - Deviations from protocol, with reasons stated | 7,9 |
|  | 11c | **Relevance and implications**  Comprehensively describe:   - Relevance of findings - Potential implications for future clinical practice and guidelines - Measures that can be taken to enhance the quality of research study - Need for and direction of future research | 8 |
| **Conclusion** | 12 | **Conclusions**   - Summarise key conclusions, in a concise and succinct manner - Outline scope for and direction of future research | 9 |
| **Additional information** | 13a | **Registration**   - In accordance with the Declaration of Helsinki*, state the unique research registration number and where it was registered, with a hyperlink to the registry entry *(this can be obtained from ResearchRegistry.com, ClinicalTrials.gov, ISRCTN etc.)* - *N.B. All retrospective studies should be registered before submission; it should be stated that the research was retrospectively registered.*   **‘Every research study involving human subjects must be registered in a publicly accessible database before recruitment of the first subject’* | 3 |
|  | 13b | **Ethical approval**   - Whether ethical approval was needed or not, stated explicitly - Reason(s) why ethical approval was/was not needed - Name of the body giving ethical approval and approval number | 3 |
|  | 13c | **Informed consent**   - State explicitly whether informed consent was obtained, or not. - State reason(s) why informed consent was/was not obtained - State the nature of consent (e.g. verbal, written, digital/virtual)* - The authors must provide evidence of consent, where applicable, and if requested by the journal - Consent should be provided for both the original intervention/procedure and publication of the study   *If consent was not provided by the patient themselves, explain why (e.g. death of the patient and consent provided by next of kin). If the patient or family members were untraceable, then document the tracing efforts undertaken | 3 |
|  | 13d | **Protocol**   - Give details of protocol (a *priori* or otherwise) including how to access it (e.g. web address, DOI etc.) - Give details of protocol registration (e.g. protocol registration number, protocol registry’s name etc.) - If published in a journal, cite and provide a full reference - If applicable, detail any amendments made to the original protocol, giving reasons why the changes were made | 3 |
| **Declarations** | 14a | **Contributorship**   - Acknowledge any patient and/or public and/or professional involvement in research - Report the extent of involvement of each contributor, specifically stating what they contributed to (e.g. patient recruitment, defining research outcomes, dissemination of results etc.). | Disclosure form |
|  | 14b | **Conflicts of interest**   - Conflicts of interest, if any, are described | Disclosure form |
|  | 14c | **Funding**   - Sources of funding (e.g. grant details), if any, are clearly stated - Role of funder stated - Guarantor named | Disclosure form |
|  | 14d | **Data sharing statement**   - Explicitly state whether or not the datasets generated during study are available on request | Disclosure form |
